# Group Contribution Estimation of Ionic Liquid Melting Points: Critical Evaluation and Refinement of Existing Models

**DOI:** 10.3390/molecules26092454

**Published:** 2021-04-22

**Authors:** Dhruve Kumar Mital, Paul Nancarrow, Samira Zeinab, Nabil Abdel Jabbar, Taleb Hassan Ibrahim, Mustafa I. Khamis, Alnoman Taha

**Affiliations:** 1 Department of Chemical Engineering, American University of Sharjah, Sharjah 26666, United Arab Emirates; dmital@aus.edu (D.K.M.); g00070704@alumni.aus.edu (S.Z.); nabdeljabbar@aus.edu (N.A.J.); italeb@aus.edu (T.H.I.); XXA732@student.bham.ac.uk (A.T.); 2 Department of Biology, Chemistry and Environmental Sciences, American University of Sharjah, Sharjah 26666, United Arab Emirates; mkhamis@aus.edu; 3 Department of Chemical Engineering, University of Birmingham, SW Campus, Birmingham B15 2TT, UK

**Keywords:** ionic liquids, melting point, property estimation, group contribution, thermal energy storage

## Abstract

While several group contribution method (GCM) models have been developed in recent years for the prediction of ionic liquid (IL) properties, some challenges exist in their effective application. Firstly, the models have been developed and tested based on different datasets; therefore, direct comparison based on reported statistical measures is not reliable. Secondly, many of the existing models are limited in the range of ILs for which they can be used due to the lack of functional group parameters. In this paper, we examine two of the most diverse GCMs for the estimation of IL melting point; a key property in the selection and design of ILs for materials and energy applications. A comprehensive database consisting of over 1300 data points for 933 unique ILs, has been compiled and used to critically evaluate the two GCMs. One of the GCMs has been refined by introducing new functional groups and reparametrized to give improved performance for melting point estimation over a wider range of ILs. This work will aid in the targeted design of ILs for materials and energy applications.

## 1. Introduction

Over the past two decades, interest in ionic liquids (ILs) has grown significantly, both in terms of academic research and industrial applications. ILs are loosely defined as salts that exist as liquids below 100 °C [1]. Their relatively low melting points result from their chemical structure; they are made of bulky, asymmetric organic cations in combination with organic or inorganic anions that do not easily form a tightly packed lattice structure [2]. ILs also exhibit several unique and interesting properties. They exhibit negligible volatility, high thermal and chemical stability, and high solubility towards a wide range of solutes. Due to the wide range of possible ILs, each with different chemical and physical properties, they are often described as “designer” liquids [3]. These characteristics make them potential candidates for various applications such as separation processes [4], heat transfer fluids [5], thermal energy storage [6], batteries [7], fuel cells [8], and tribology, both as lubricants [9] and lubricant additives [10], and even space technology [11]. Improvements in IL synthesis technology in recent years has also facilitated their large-scale production [12]. Today, there are multiple manufacturers with the ability to mass produce ILs [12,13].

Melting point is one of the fundamental defining characteristics of ILs and also a key physical property in selecting and designing ILs for materials and energy applications. For example, the use of ILs as solvents or heat transfer fluids in industrial processes requires ILs that remain in the liquid phase over the full range of process conditions involved. Melting point defines the lower limit of the liquidus range of ILs, with the upper limit usually defined by the decomposition temperature. In addition, the application of ILs as phase change materials for thermal energy storage necessitates the selection of an IL with a specific melting point suited to the temperature at which thermal energy absorption or release is required [14].

One of the most important characteristics of ILs is their “designer” nature. IL properties can be tuned by varying the cation-anion combination, changing the hydrocarbon chain length on the cation, or by adding various functional groups. It has been estimated that there are in the order of 10^6^ possible ILs, yet only a few thousand have actually been synthesized and characterized. [15,16]. This vast number of possible ILs means that it should be possible, in theory, to design ILs with optimized properties for any selected application. In practice, however, this does not happen, due to the obvious cost and time constraints. To resolve this issue and allow ILs to truly become designer liquids, predictive models are an essential component in the screening of the almost limitless array of possible ILs.

Models for estimating physical properties can be categorized as either theoretical, semi-empirical or empirical [17]. Theoretical methods, such as molecular modeling, are derived from first principles and are usually extremely computationally intensive. They are most useful for developing a fundamental understanding of physical property behavior on a molecular level, but are usually impractical for large scale computational screening of molecules. On the other end of the scale, empirical methods are fast and easy to apply, and are useful for interpolation when sufficient data already exists. However, they are significantly limited in their predictive capabilities. The semi-empirical method is the most useful approach when predictive modelling and efficient screening of large numbers of molecules is required. These models use previously regressed parameters in combination with an equation that models the relationships between the structure of the molecule and its physical properties.

There are primarily two semi-empirical approaches available for predicting IL properties: group contribution methods (GCMs) and quantitative structure property relationship (QSPR) models. QSPR models use the physical, chemical, or physicochemical properties as descriptors, upon which the predictive mathematical model is built [18]. QSPR models mainly rely on quantum calculations to attain the values of the various descriptors used for predicting the IL properties [19]. On the other hand, GCMs mainly use the frequency of occurrence of functional groups within the chemical structure for calculating properties [17]. The fundamental assumption in GCMs is that the physical property of the whole molecule can be represented by the additive contribution of its functional groups. The key advantage of this approach is that all the information needed to use the model is available in the chemical structure. There is no need to make any experimental measurements or perform quantum calculations before applying the GCM for property estimation. For many engineering applications, such as process simulation or computer-aided molecular design, GCMs are preferable to QSPR models, as they are less computationally intensive and are more flexible in their application. In the past decade, GCMs have been developed to estimate IL thermodynamic and physical properties such as melting point [19,20,21], density [22,23,24,25,26,27], viscosity [28,29,30,31], heat capacity [32,33,34,35,36], and thermal conductivity [37,38,39]. However, most of these GCMs were developed several years ago and are based on different and limited datasets. As a result, it is meaningless to compare their reported statistical measures, such as coefficient of determination (R^2^) or average absolute relative deviation (AARD), since these are not calculated against the same test sets. Furthermore, since the models were developed at a time when significantly fewer ILs had been characterized experimentally, the group contribution parameters available in such models are insufficient for property prediction of many new types to ILs. This presents a major problem in selecting an appropriate GCM for future use. In recent years, a wealth of experimental data has been published; therefore, it is necessary to revisit, reevaluate, improve, and extend such models.

GCMs have been used for property prediction extensively since the 1980s. One of the earliest models was developed by Joback and Reid [40], which estimated a wide variety of thermophysical properties of pure molecular compounds and greatly expanded on the work done by Lydersen [41]. Group contribution models are based on the fundamental assumption that the property of a compound depends upon the type and quantity of functional groups that the compound is composed of. Each functional group makes a certain contribution towards the overall physical property of the compound. In the simple GCMs, these contributions are assumed to be additive; the frequencies of occurrence of each functional group are multiplied by their contribution parameters and added together to obtain the property for the whole compound [40]. More advanced GCMs involve more complex procedures for obtaining the overall property from the individual functional group parameters; for example, they may have parameters to represent the interaction between functional groups, thereby accounting for the dependence of the physical property on such interactions. The main advantages of the GCM approach are its ease of application, breadth of applicability, low computational cost, and its reliance only on structural information without the need for more complex parameters. This is in contrast to the QSPR approach, for example, which often relies upon descriptors derived from quantum calculations or other experimentally measured physical properties. It is also comparatively easy to understand the physical meaning of the parameters in GCMs, as well as extend the applicability of these models by estimating the values for new groups that might not have been incorporated into the initial model. Finally, group contribution models have the benefit of being extendable to work for multiple properties while using the same structural building blocks. Depending on the property being modeled, the fundamental equation for the group contribution models may remain the same, while the group contribution parameters will be regressed according to the new property. Alternatively, the same building blocks can be used, but with new parameters and a new equation more suited to the new property. This greatly reduces the setup time for modeling various properties and provides a consistent platform to build on. Furthermore, often several GCMs are needed to work together for molecular design, since several physical properties are important in the design of molecules for specific applications. Having several GCMs that use the same structural building blocks facilitates their incorporation into an integrated design framework.

Many researchers have developed models for the estimation of IL melting points. Table 1 provides a summary of the models reported in literature. It was observed that they generally fall under the categories of QSPR or GCM, with a recurrent neural network (RNN) also included. However, many of the models are limited to specific families of ILs, such as imidazolium halides, for example. Such models have a low range of applicability and are of very limited use in computer-aided molecular design (CAMD) of ILs for different applications. In all cases, the number of compounds used in the test set and training set is lower than the currently available data for IL melting points, often by a significant margin. Therefore, significant scope exists to build upon this previous work by compiling an updated database of IL melting point data, and using it to test, update, and extend the models.

Typically, two statistical measures are used in testing the accuracy of such models: coefficient of determination (R^2^) and average absolute relative deviation (AARD). As seen in Table 1, some authors reported R^2^, others reported AARD, and a few reported both. Therefore, it is difficult to decide which model is performing the best, since these different statistical measures have been used. This is further compounded by the fact that different test sets, with different numbers of unique ILs, have been used in calculating the statistical measures. A fair comparison can only be made when using the same test set of data, which should be as comprehensive as possible.

It should also be mentioned that there are several difficulties in determining the melting points of ILs which, thereby, can impact the accuracy of data-driven predictive models. Valderrama and Campusano [55] summarized some of the key issues in two major areas: glass formation and multiple transitions. While cooling, there is the possibility of incorrect measurement due to the IL going through glass transition, where the kinetics are much slower. The difficulties in performing these experiments and estimating the melting point has somewhat reduced with the advent of more advanced Differential Scanning Calorimetry (DSC) machines. Conventional DSC machines suffered from various issues like insensitive sensors, requirement of external heat, inability to achieve constant cooling rates, and controlling drift in the temperature curves [56]. Most modern DSCs have rectified these errors, by introducing more thermocouples and heating elements, as well as measuring for any variations across the chamber, and accompany these with more optimized software and control mechanisms [57]. However, there are still aspects of the experimental process that can lead to errors, such as contamination of samples, humidity, formation of various liquid and plastic phases, pressure variations, thermal stability issues, and calibration and user errors [58]. There is also a lack of a universal testing protocol for such measurements, from material handling to temperature ranges and rates of change during the heating and cooling process, which can cause issues. Yamamoto [59] outlined these issues, and demonstrated the challenges in building a reliable predictive model as a result of the variance in the limited amount of experimental data available. Such issues lead to a certain amount of “noise” in the data. As long as the errors are randomly distributed about the mean, then this is not a major issue when applying regression-based models. Since GCMs are developed via parameter regression, the best fit parameters will be obtained by minimizing an objective function, such as the sum of the squares of residuals, for example. Random errors on the positive and negative side will effectively balance each other during regression to reach the best fit values of the parameters. However, the statistical measures used to test the models will also include the experimental error resulting from test set of data, and the measured performance of the model in terms of R^2^ or AARD cannot, therefore, be better than that level of experimental error present within the test set.

As more melting point data has been gathered on a variety of ILs containing different cation and anion groups, it has become possible to develop more extensive models. The last two models in Table 1 are the most generic GCMs available today for IL melting point prediction and, thus, are the most useful for IL design for materials and energy applications. Therefore, we have selected these two GCMs for further detailed analysis in this work. Herein, the model by Lazzús [20] is referred to as GCM1 and the model by Gharagheizi et al. [21] is referred to as GCM2.

## 2. Results and Discussion

### 2.1. Database Development and Refinement

The new IL melting point database (see “Methodology” section) was used to evaluate the performance of the two selected group contribution models, GCM1 and GCM2. The preliminary database (DB-P) of raw data was initially compiled from a range of literature sources and the ILThermo website, without any data cleaning. However, it was noticed there were some issues that needed to be resolved before the database could be used for testing the selected GCMs. There was some duplication of datapoints that would cause undue weight to go towards those literature sources. Therefore, the database was carefully checked, and any duplicates were eliminated. The resulting refined database (DB-R) contained around 1500 datapoints.

Ideally, it would be possible to test both models against DB-R. However, due to limitations in each model resulting from the lack of certain required group contribution parameters, this was not possible. For example, the imidazolium functional group included in GCM1 was only designed to use substitutions on its 1st, 2nd, or 3rd positions. As such, it was not possible to represent the structures of ILs containing substitutions in other positions, such as 1,2,4-trialkylimdazolium ILs. In other cases, it was not possible to build the IL due to the lack of certain functional groups in each model. For example, metallic salts and ILs with unusual ring structures, such as benzotriazolium, could not be modeled in GCM1. Similarly, ILs containing the azido group were not represented by the functional groups in GCM1. The next necessary step, therefore, was to further reduce the database to include only those ILs for which each GCM could estimate the melting point. This resulted in a new, smaller database, DB-1, which was used for the analysis and comparison. It contains only GCM1- and GCM2-compatible data, consisting of 1305 datapoints, with 933 unique ILs made from 484 unique cations and 94 unique anions.

### 2.2. Model Evaluation and Comparison

Comparison of the two models begins with a qualitative analysis of the similarities and differences between their respective approaches. For GCM1, the cation is assumed to consist of a core group, such as imidazolium, pyridinium, or phosphonium, for example. Each cation core group has a number of available locations for attaching other functional groups. Imidazolium, for example, has three available locations: the two nitrogen atoms, and the carbon atom between the nitrogen. Smaller subgroups, such as -CH_2_- or -CH_3_, can be added to construct the alkyl chains typically present in IL cations. The model also allows for the addition of other functional groups, such as -OH. The anion is constructed from smaller subgroups, such as -SO_2_ and CF_3_, for example. Deconstructing the IL into its functional groups for the purpose of applying the GCM1 model is relatively straightforward. The functional groups used within the model are representative of the typical structures seen in commonly used ILs and, therefore, the application of the model is intuitive. In contrast, GCM2 is significantly more complicated to implement. The first issue is the larger number of functional groups that are used in GCM2; there are 67 functional groups in GCM1 compared with 80 groups in GCM2. Furthermore, the rules involved in deconstructing the IL into its functional groups using GCM2 are highly complex. It takes a much longer time to map each IL using GCM2 and there is a much greater chance of making an error due to several overlapping functional groups. Therefore, great care must be taken when implementing GCM2.

**Figure 1:**
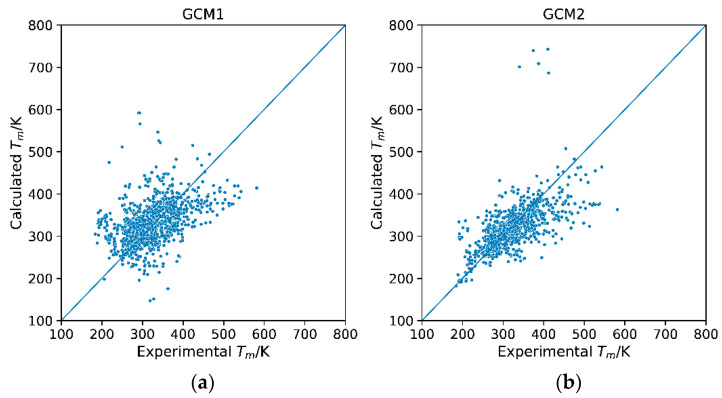
Parity plots of calculated versus experimental melting points for (**a**) GCM1 and (**b**). GCM2.

After the development of the most extensive database possible for testing both models, DB-1, the next step was to use GCM1 and GCM2 to estimate the melting points for every IL in the database. This involved breaking down and mapping out the IL structure in terms of its functional groups for each GCM, as illustrated in the “Methodology” section. Subsequently, the mapping for each IL was imported into the Python DataFrame and then each model was implemented to estimate the melting points for every ionic liquid in DB-1. The calculated melting points were then compared with the experimentally measured melting points. The parity plots of calculated versus experimental melting points for each model are shown in Figure 1. Ideally, the data points should follow the diagonal line, indicating that the calculated and experimental values are the same. However, we observe in both cases, the data is spread significantly. GCM2 performs overall somewhat better than GCM1, with its data points more tightly packed around the 45° line. However, for GCM2, there is a cluster of points which lies very far from the y = x line. These outliers will be discussed in more detail later. The average absolute relative deviation (AARD) was also calculated as a statistical measure of the performance of each model. For GCM1, the AARD was 13.98% while for GCM2, the AARD was 9.66%. This lower AARD indicates that GCM2 performs better than GCM1 overall; however, both models performed significantly worse when tested against the new database compared with their AARD values reported in literature using their original smaller and less diverse test datasets (Table 1).

There are several possible reasons why GCM2 performs better than GCM1. Firstly, the model was created based on a much larger training set of IL data; GCM1 had a training set of 200 IL datapoints, whereas GCM2 used a broader training set of around 400. Therefore, we would expect the parameters of GCM2 to be more robust and perform better when tested against the larger database. Secondly, the smaller functional groups used in GCM2 appear in a much broader range of ILs and, therefore, each functional group gets trained by a much wider range of ILs than those used in GCM1. This helps to improve the robustness of the model for application towards a wider range of ILs. Thirdly, the functional group definition for GCM2 was based on statistical significance, whereas, in GCM1, the functional groups were selected intuitively based on the typical structures observed in ILs. The use of statistical approach to guide functional group selection could be a significant factor in the overall better performance of GCM2.

**Figure 2:**
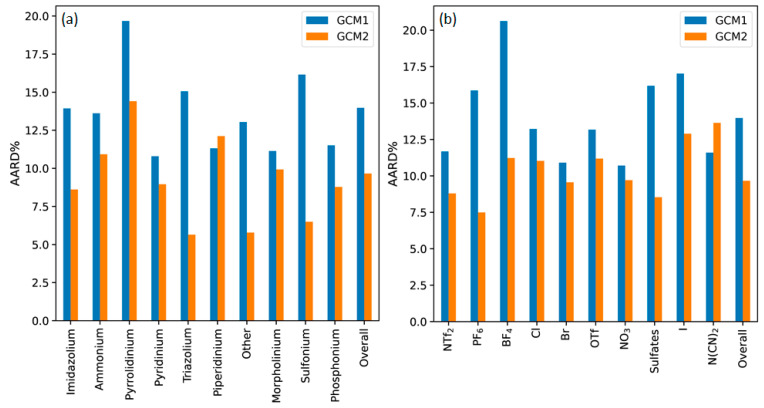
AARD% for each model according to (**a**) cation type and (**b**) anion type.

To further analyze the performance of each model, the percentage AARD values for each cation type and anion type were determined, as illustrated in Figure 2. The general trend was that GCM2 exhibited a lower AARD than GCM1, except for piperidinium among the cations, and N(CN)_2_ among the anions. This may be due to the fact that there are relatively few data points for ILs containing these types of cations and anions. Also, in the case of N(CN_2_), the high error can be attributed to the existence of CN groups in both N(CN)_2_ and B(CN)_4_ anions. The contribution from CN towards *T_m_* within B(CN)_4_ was much larger than that in N(CN)_2_. This may be due to symmetrical tetrahedral structure of B(CN)_4_, which tends to cause an increased melting point. By contrast, N(CN)_2_ is linear in orientation, which results in lower melting point. However, when the models were parametrized, CN was given equal weighting in both ions. This may have introduced a larger error in the regressed parameters of GCM2. The biggest differences between the performance of GCM1 and GCM2 occurred for the “other” category among the cations, which incorporates all those ILs containing cations that are not among the most frequently occurring cation types. Among the anions types, BF_4_ exhibits the biggest difference. GCM1 is significantly worse for ILs containing BF_4_ and PF_6_, two of the most common anions found in ILs. This may be explained by the large difference between the number of BF_4_ and PF_6_ data points used to train each model. For GCM2, 70 data points for PF_6_-based ILs and 56 data points for BF_4_-based ILs were used in the training set. By comparison, only around 12 data points for PF_6_-based ILs and 18 data points for BF_4_-based ILs were included in the original training set of GCM1. Consequently, we would expect the relevant parameters for GCM2 to be much more accurate than those in GCM1.

**Figure 3:**
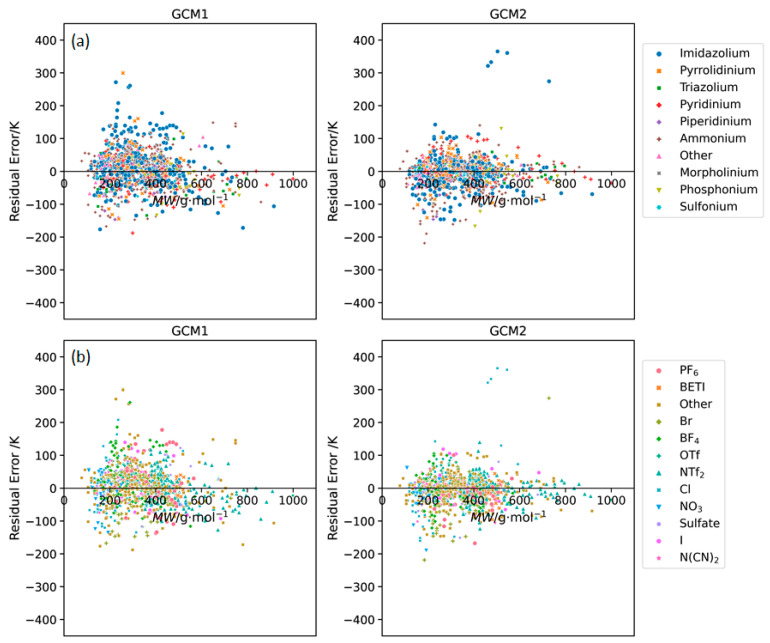
Residual plots of error versus IL molecular weight obtained using GCM1 and GCM2 to estimate melting points for ILs containing the major (**a**) cation and (**b**) anion types.

The residual plot is an important tool for the interpretation of the errors present in predictive models. The most desirable models are those where the residuals are small and evenly distributed above and below the x-axis. The presence of any other trend in the residual plot could be indicative of systematic error in the model. Figure 3 shows the residual plots of error versus melting point for GCM1 and GCM2. In both cases, the data is relatively evenly distributed on the positive and negative sides of the residual plot. However, it is apparent that there was a slight trend in the data for both models, where the residuals were slightly more on the negative side, i.e., slightly more points below the x-axis, at low MW (<250 g∙mol^−1^), and more on the positive side at medium MW (250 to 400 g∙mol^−1^), then they become more negative again at high MW (>400 g∙mol^−1^). This indicates that, to some extent, both models inaccurately represented the relationship between IL melting point and MW.

**Figure 4:**
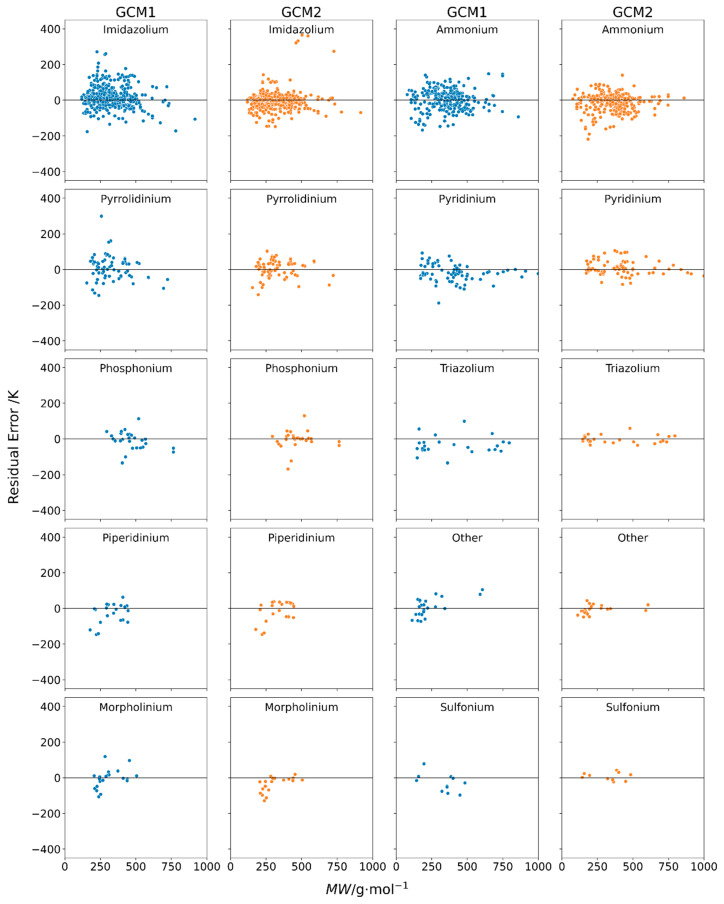
Individual residual plots of error versus molecular weight using GCM1 (blue) and GCM2 (orange) to estimate melting points for ILs containing several different cation types.

By examining the residual plots for different subcategories of IL melting point data, we can gain a better understanding on whether the model is performing adequately in each case. Figure 4 shows the individual residual plots for different cation types. For most cation types, we observed that the performance of both GCMs depends somewhat on the molecular weight of the IL. In many cases, at low MW, the error was negative, indicating that the model was underestimating *T_m_*. In the mid-range MW, there tended to be more overestimation of *T_m_*, and then for higher MW ILs, again there was more underestimation of *T_m_*. This can be explained by the fact that both GCMs are additive in nature. That is, each functional group makes a fixed contribution towards the melting point. For example, in GCM1 the -CH_2_- functional group had a contribution of 4.26 K, whereas, for GCM2, the -CH_2_- contribution was 1.62 K. Since these contribution parameters are positive, each model assumes that every additional -CH_2_- group added to the cation alkyl chain raises the melting point by that value. However, it has been well reported [60] that the melting points of ILs tend to decrease with increasing alkyl chain length until a minimum is reached at around 6 to 8 carbons, following which the melting point increases with increasing alkyl chain length. With increasing chain length, melting points decrease initially due to the increasing entropy. However, for larger chains, the Van der Waal’s interactions play a more dominant role, thus resulting in an increase in the melting point. These effects are further exacerbated with the energy differences due to changing bond and dihedral angles while undergoing phase change, creating a greater energy requirement and increasing the melting point [60]. Therefore, the additive nature of these GCMs is preventing each model from accurately representing the observed complex relationship between alkyl chain length and melting point. This explains why, at low MW, when the alkyl chains tend to be shorter, we observe that both GCMs tend to underestimate *T_m_*. The additive nature of the GCM predicts that the melting point will be lowest for ILs with very short alkyl chains when, in fact, it tends to relatively high compared with those having medium alkyl chain length.

Among the various cation types, the largest spread is observed in imidazolium and ammonium. This is indicative of the very large number of data points available for these types of cations with a variety of counter anions.

**Figure 5:**
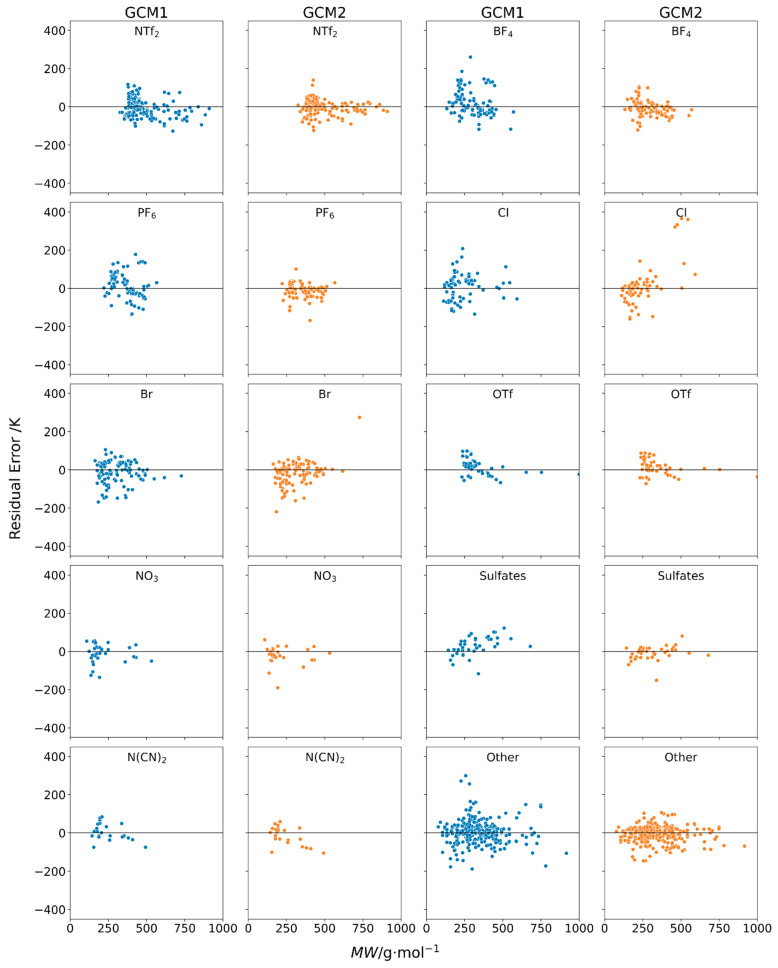
Individual residual plots of error versus molecular weight using GCM1 (blue) and GCM2 (orange) to estimate melting points for ILs containing several different anions.

Figure 5 shows the individual residual plots for different anion types. Again, we observe the trend that both models tend to underestimate *T_m_* for low MW ILs, then overestimate for medium MW ILs, and again, underestimate for high MW ILs. Although NTf2 is the most frequent anion in the database, the greatest spread is observed in BF_4_ and PF_6_ for GCM1, whereas for GCM2, Cl based ILs have the largest spread. The reasons for this are discussed later.

Overall, GCM2 has been shown to outperform GCM1 based on the statistical measure, AARD. However, accuracy is only one of the desirable characteristics of a GCM. It is also important that the model should be easy to implement for the estimation of the physical property; furthermore, the model should be easy to implement within process simulation software and computer aided molecular design frameworks. This is where GCM2 has significant disadvantage compared with GCM1. As mentioned previously, it is difficult and time consuming to map out the structures of ILs for implementation of GCM2. Furthermore, due to the complexity of the functional group rules, it would be extremely difficult to combine this approach with structural constraints in a CAMD framework to allow the design of structurally feasible ILs. Another issue with GCM2 is the fact that, if a group or substructure is not present in the mapping table, its contribution is assumed to be zero. For example, the hexafluorotantalum and hexafluoroantimonate anion groups have the same contribution, as the model does not have either the tantalum or antimony groups. Although ammonium-based groups can be mapped out easily as a result of the availability of the N^+^ group, there is no such availability of a P^+^ group for phosphonium-based anions. This will inevitably lead to extrapolation of GCM2 beyond its reasonable range of applicable ILs and give the user a false sense of security. To further add to the problems of the mapping process, it is very difficult to generate the structural map from a holistic perspective taking into account the mapped data already available for other ILs. In conclusion, although GCM2 is more accurate, it comes at a cost to its utility as a quick and simple estimation tool, which is one of the main purposes of a GCM.

### 2.3. Refinement and Reparametrization of GCM1

While GCM1 has several advantages, including ease of use and applicability towards simulation software and CAMD frameworks, it displays significantly lower accuracy than GCM2. As discussed previously, this may be mainly attributed to the very small training set that was used to regress its group contribution parameters. Therefore, given that a much larger database has been compiled in this work, it was decided to further refine and reparametrize GCM1 to establish whether it can achieve an AARD comparable to that of GCM2. Firstly, some new functional groups were created to solve the problems identified in the model. New anion groups for BF_4_, PF_6_, B(CN)_4_, oleate, and phosphate were created, since these were identified as structures for which original GCM1 was not giving adequate results. In addition, a new cation group (>P = O) was created to differentiate this functionality from the existing phosphonium group. After remapping the relevant ILs in the database according to these new functional groups, the model was fully reparametrized using Keras library in Python. The database was split into two separate parts: 50% of the data was used as the training set, the other 50% was used as the test set. The parameters were regressed by minimizing the objective function, MAE, via stochastic gradient descent (SGD). The new optimized parameter values for the cation functional groups and anion functional groups are shown in Table 2 and Table 3, respectively. The reoptimized model, herein referred to as GCM1-R, demonstrated an AARD of 9.75% when tested against the test set, and 9.67% against the whole database, bringing it in close agreement with the AARD for GCM2.

**Figure 6:**
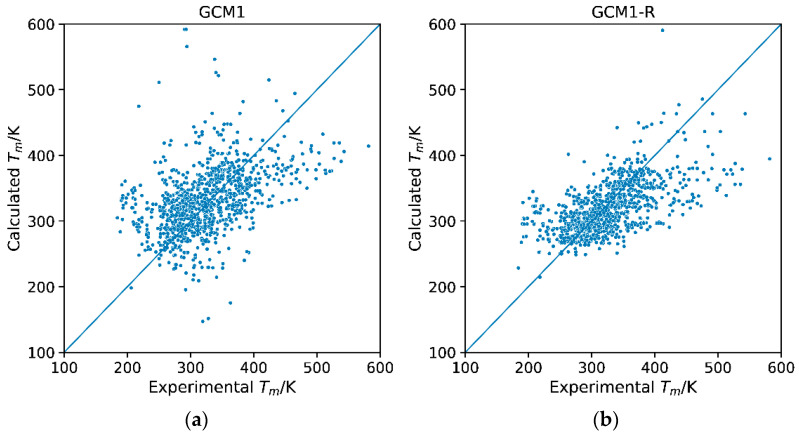
Parity plots of calculated versus experimental melting points for (**a**) GCM1 and (**b**) GCM1-R.

The parity plot for the reparametrized model, GCM1-R, is compared with the parity plot for GCM1 in Figure 6. The significant improvement in the model performance can be observed, with the data points for GCM1-R more tightly packed around the y = x line. It is also notable that there was a significant reduction in the number of major outliers. One of these relates to the icosahedral anion, which had six bromide groups attached. Due to the large change in the weight of the -Br parameter after reparametrization, and the heavy influence of the -Br parameter in this IL, we observed a significant increase in the predicted melting point. This drastic change in the weighing of the bromide group in the overall model had the consequence of affecting the predicted melting point of the “hexabromide 1-carbon icosahedral” ionic liquid. The lack of data for multiple bromine-containing anions resulted in this datapoint having an error of around 200 K between the observed and predicted melting points of the sole datapoint. These groups were also extremely large and require multiples of various subgroups, mainly optimized for single occurrences in the overall IL. As a result, the overall error was compounded.

**Figure 7:**
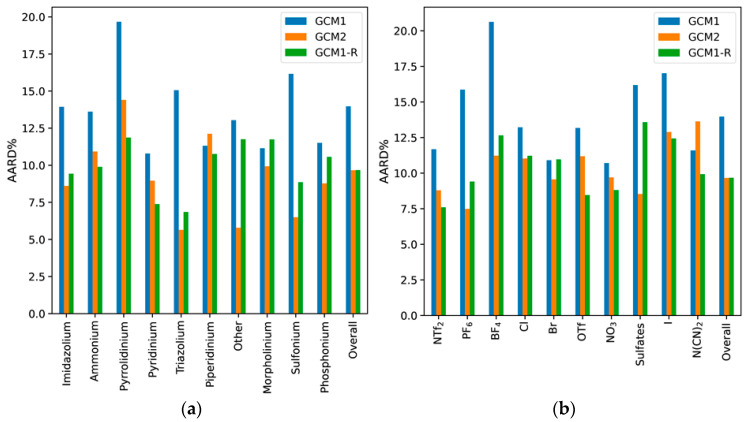
Comparison of AARD for GCM1-R with GCM1 and GCM2 for the estimation of IL melting point according to (**a**) cation type and (**b**) anion type.

The performance of GCM1-R for estimating the melting points of different types of cations and anions is shown in Figure 7. In comparison with GCM1, we see improvements for most types of cations and anions. Compared with GCM2, the performance was similar for many types of cations and anions; however, GCM2 was still performing better for ILs containing the “other” category of cations, and ILs containing sulfate-based anions. This can be attributed to the method used to model such groups. GCM2 was built using groups of statistical significance relative to its database, which accounted for most trends and behaviors expected from the various cation and anion groups. The error, therefore, resulted from the uncertainty present in the less statistically or homologically significant groups. Since GCM1-R is derived from the original GCM1, it still suffers from some of its disadvantages. In the case of the “other” groups, the model uses more generalized subgroups that can be applicable to more datapoints, thereby trying to reduce overall error at the expense of accuracy in prediction. This also applies to the sulfate ions, which have functional groups in common with the sulfonyl-imide anions (NTf_2_, BETI, OTf), which have been studied more intensively, and consequently have more datapoints in the overall dataset. This results in the SO_2_ group being more optimized towards these groups, resulting in a higher error for the sulfates.

**Figure 8:**
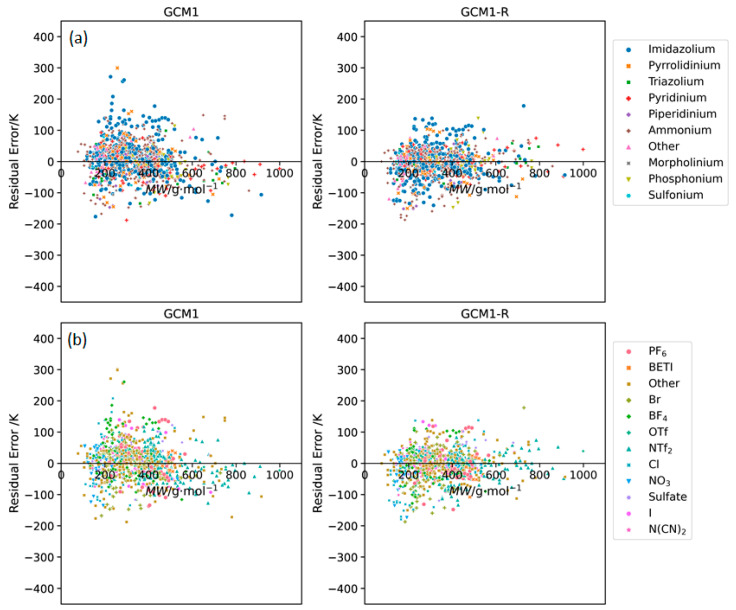
Residual plots of error versus IL molecular weight obtained using GCM1 and GCM1-R to estimate melting points for ILs containing the major (**a**) cation and (**b**) anion types.

Figure 8 shows the residual plots of error versus MW for GCM1 and GCM1-R. Again, the improved performance of GCM1-R over GCM1 can be clearly seen. However, the same trend is still apparent; for ILs with low MW, the model underestimates *T_m_*, for ILs with medium MW, the model overestimates *T_m_*, and for high MW ILs, the model underestimates *T_m_*. As explained previously, this is a problem that is inherent in additive GCM approach and cannot be overcome by new group definition or reparametrization. However, overall, it has been demonstrated that GCM1-R gives equivalent performance to GCM2 in terms of AARD, while having significant advantages in terms of ease of use, and applicability to simulation software and computer-aided molecular design frameworks.

## 3. Methodology

As a basis for testing and extending the selected GCM models, an Excel database of IL melting point data was compiled (see Supplementary Materials) from a variety of literature sources, including the NIST ILThermo database [61], a physicochemical properties reference book for ILs [62], and individual journal papers [14,45,51,63,64,65,66,67,68,69,70,71,72,73,74,75,76,77,78,79,80,81,82,83,84,85,86,87,88,89,90,91,92,93,94,95,96,97,98,99,100,101,102,103,104,105,106,107,108,109,110,111,112,113,114,115,116,117,118,119,120,121,122,123,124,125,126,127,128,129,130,131,132,133,134,135,136,137,138,139,140,141,142,143,144,145,146,147,148,149,150,151,152,153,154,155,156,157,158,159,160,161,162,163,164,165,166,167,168,169,170,171,172,173,174,175,176,177,178,179,180,181,182,183,184,185,186,187,188,189,190,191,192,193,194,195,196,197,198,199,200,201,202,203,204,205,206,207,208,209,210,211,212,213,214,215,216,217,218,219,220,221,222,223,224,225,226,227,228,229,230,231,232,233,234,235,236,237,238,239,240,241,242,243,244,245,246,247,248,249,250,251,252,253,254,255,256,257,258,259,260,261,262,263,264,265,266,267,268,269,270,271,272,273,274,275,276,277,278,279,280,281,282,283,284,285,286,287,288,289,290,291,292,293,294,295,296,297,298,299,300,301,302,303,304,305,306,307,308,309,310,311,312,313,314,315,316,317,318,319,320,321,322,323,324,325,326,327,328,329,330,331,332,333,334,335,336,337,338,339,340,341,342,343,344,345,346,347,348,349,350,351,352,353,354,355,356,357,358,359,360,361]. This initial database contained 2061 data points from over 300 literature sources. Preliminary data cleansing was performed to remove duplicated data points and convert all IL synonyms to a single common name. For example, the anion ‘bis((trifluoromethyl)sulfonyl)imide’ was present in the database in 7 different ways, using a combination of different brackets and naming methods.

Following the initial data cleansing in Excel, the database was imported into Python as a DataFrame using the Pandas package [362]. The resulting DataFrame was then used as the basis for data segmentation and analysis. The seaborn [363] and matplotlib [364] packages in Python were used for generation of the various data visualizations.

In a separate Excel file, the various cations and anions were deconstructed and described in terms of the functional building blocks used for each model. This GCM mapping was subsequently imported into Python and merged with the existing DataFrame containing the experimental data points. GCM1 and GCM2 were implemented within Python to determine the corresponding calculated melting point for each experimental data point in the database.

**Figure 9:**
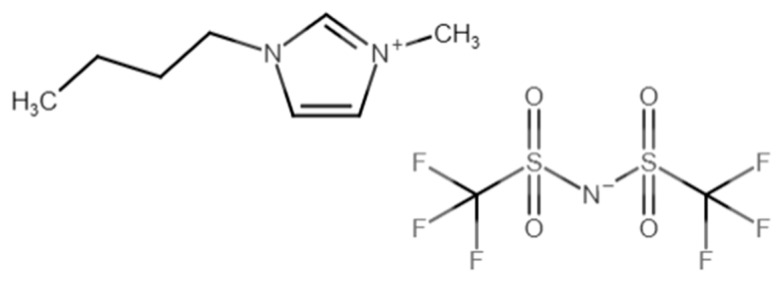
Chemical structure of 1-butyl-3-methylimidazolium bis((trifluoromethyl)sulfonyl)imide.

Each of these models is linear and uses the same fundamental equation for estimating the melting point of the IL:

(1) $$T_{m}=T_{0}+\Sigma n_{i}\Delta T_{m,i}$$

where *n_i_* represents the frequency of occurrence of functional group, *i*, Δ*T_mi_* represents the contribution parameter of group, *i,* and *T_0_* is a global fitted parameter. For the GCM1, *T*_0_ is 288.7 K, while for GCM2, *T*_0_ is 264.29 K. An illustration of the procedure involved in applying each model for the estimation of one IL, 1-butyl-3-methylimidazolium bis((trifluoromethyl)sulfonyl)imide, is given in Table 4. The chemical structure of this IL is shown in Figure 9. The cation and anion functional groups needed to construct this IL via GCM1 and GCM2, along with the frequency of occurrence and the associated contribution parameters, are shown in the table. For each group, the frequencies of occurrence are multiplied by the contribution parameters and the products are added along with the global parameter to obtain the estimate of the melting point. Further details on the functional group building blocks and their rules of application for GCM1 and GCM2 can be found in Lazzús [20] and Gharagheizi et al. [21], respectively.

For reparametrization of existing parameters and regression of new group parameters, the Keras package [365] was used in Python. The database was split into a training set and test set, each containing 50% of the available data. A single dense layer was created in Keras using the model parameters as the input and the mean absolute error (MAE) was used as the loss function. Optimization was performed using stochastic gradient descent (SGD).

Absolute average relative deviation (AARD) was used as the statistical measure of the performance of the models and was calculated according to the following equation:

(2) $$\mathrm{ARD}\left(\%\right)=\frac{1}{n}\overset{n}{\underset{i=1}{\sum }}\left|\frac{T_{m,calc}-T_{m,exp}}{T_{m,exp}}\right|\times 100\%$$

## 4. Conclusions

Over the past few decades, the GCM approach has been used extensively in process simulation and CAMD software, due to its reasonable accuracy, relative ease of use, wide range of applicability, and ease of integration into CAMD frameworks. In this paper, two of the most comprehensive GCMs for the estimation of IL melting points were critically examined. An up-to-date, comprehensive database of IL melting point data was compiled. The IL structures were mapped according to the functional group building blocks used for each GCM. The two GCMs were implemented against the full database, and the performance of each model was assessed based on its AARD for various categories of ILs. Both models were found to yield higher AARDs than those reported in the original papers. This is due to the much larger and more comprehensive test set used in this study. Both GCMs were also found to exhibit a certain amount of systematic error when examining the relationship between error and IL MW. This was attributed to the additive approach used in each model, that is unable to accurately represent the more complex relationship between the size of an IL and its melting point. It was found that GCM2 performed significantly better than GCM1 based on the statistical measure, AARD. However, due to the complexity of the functional groups used in GCM2, there is a high likelihood of user error, and it does not lend itself towards facile integration with structural constraints into a computational framework for IL design. On the other hand, GCM1 has straightforward functional building blocks that are simple and intuitive to use for IL melting point estimation. The fact that GCM1 was trained against a much smaller dataset than GCM2 was identified as a major reason for its significantly higher AARD. Furthermore, some problems with the functional group definitions, leading to increased error, were identified. To address these issues, several new functional groups were defined, and the whole model was reparametrized against a much larger training set than that used in the original model. The refined and reparametrized model, GCM1-R, was found to perform significantly better than the original GCM1, yielding a similar AARD to that obtained with GCM2. In combination with its ease of use and breadth of applicability, it is anticipated that this refined GCM will aid in the design of ILs for materials and energy applications, by reducing the need for extensive synthesis and experimental characterization of new ILs.

## Figures and Tables

**Table 1:** Summary of existing models for IL melting point estimation.

Work	Type of Model	Applicability	Number of ILs Used	R^2^	AARD
Huo et. al. [42]	GCM	Imidazolium/Benzimidazolium	190	0.8984	5.86%
Aguirre et. al. [43]	GCM	Diverse	136		7.8%
Chen et. al. [44]	GCM	Diverse	111		4.7%
Katritzky et. al. [45]	QSPR	Imidazolium/Benzimidazolium bromides	149	0.7442/0.7517/0.6899	
Katritzky et. al. [19]	QSPR	Pyridinium bromides	126	0.788/0.713	
Trohalaki and Pachter [46]	QSPR	Triazolium bromides, Triazolium nitrates	26	0.8730/0.8390	
Farahani et. al. [47]	QSPR	Diverse	805	0.721	7.3%
Bini et. al. [48]	RNN	Pyridinium bromides	126	0.872	
Carrera and Aires-de-Sousa [49]	QSPR	Pyridinium bromides	126	0.933	
Zhang et. al. [50]	QSPR	Imidazolium tetrafluoroborates, Imidazolium hexafluorophos-phates	41	0.776/0.842	
Eike et. al. [51]	QSPR	Ammonium Bromides	75	0.790/0.775	
Varnek et. al. [52]	QSPR	Diverse Bromides	717	0.61	
López-Martin et. al. [53]	QSPR	Imidazolium	62	0.8690	
Yan et. al. [54]	QSPR	Imidazolium bromides, Imidazolium chlorides	50	0.8900	
Lazzús [20]	GCM	Diverse	400	0.8841	7.07%
Gharagheizi et. al. [21]	GCM	Diverse	799	0.811	5.82%

**Table 2:** New and reparametrized cation functional group parameters for GCM1-R.

Group, *i*		Δ*T_m,i_*/K
*T*_0_		210.97
Imidazolium	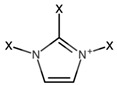	6.00
Pyridinium	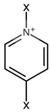	59.09
Pyrrolidinium	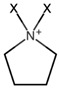	50.66
Pyrrolidinonium	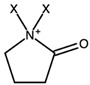	0.07
Piperidinium	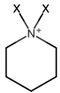	48.26
Morpholinium	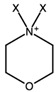	39.74
Ammonium	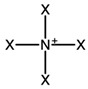	−4.38
Sulfonium	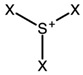	5.00
Phosphonium	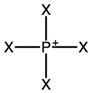	−10.01
Triazolium	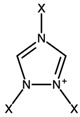	31.15
Pyrazolium	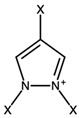	0.11
Thiazolium	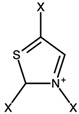	−0.19
-H		24.52
-CH_3_		30.98
-CH_2_-		−0.22
-CH<		−8.28
-OH		44.86
-O-		−18.99
>C<		12.71
-COO		9.88
-CN		40.01
-NH_2_		35.07
-F		22.38
-Br		22.24
-Cl		0.11
-SO_2_-		5.95
-Benzyl		52.26
-Vinyl		0.86
-CF_2_-		10.05
-CF_3_		30.22
-SF_5_		−5.79
>P = O		−36.95

**Table 3:** New and reparametrized anion functional group parameters for GCM1-R.

Group, *i*	Δ*T_m,i_*/K
-CH_3_	1.55
-CH_2_-	−2.71
>CH-	1.06
>C<	−0.77
>CO	−7.63
-COO-	−20.94
-HCOO	−24.97
-OH	−0.72
-O- [-O]	2.67
-CN	−16.50
-N- [>N-]	−3.87
-NO_3_	9.21
-S-	0.42
-SO_2_-	−4.82
-CF_3_	−10.06
-CF_2_-	4.27
-F	−4.74
-Cl	26.16
-Br	50.64
-P	4.77
-B	−11.05
-I	31.77
-Al	−5.92
-As	6.27
-Nb	−2.74
-Ta	0.25
-Sb	12.00
-Sn	0.23
-W	−0.28
-CB_11_H_11_- (carborane)	18.88
-CB_11_H_12_ (carborane)	18.19
-CB_11_H_6_ (carborane)	−16.68
CH- (ring)	0.18
=C< (ring)	17.02
>CO (ring)	3.72
-O- (ring)	3.34
PF_6_	28.32
BF_4_	1.18
B(CN)_4_	−8.80
Oleate	−12.14
Phosphate	18.23

**Table 4:** Estimation of the melting point of ILs using GCM1 and GCM2 with 1-butyl-3-methylimidazolium bis((trifluoromethyl)sulfonyl)imide as an illustration of the general procedure.

**GCM1**				
**Type**	**Group*, i***	***n_i_***	**∆*T_m,i_/K***	***n_i_*∆*T_m,i_/K***
**Cation**	Imidazolium	1	22.837	22.837
	CH_3_	2	−4.384	−8.768
	CH_2_	3	−3.759	−11.277
	H	1	−20.98	−20.98
**Anion**	–N– [>N–]	1	−113.15	−113.15
	SO_2_	2	114.17	228.34
	CF_3_	2	−51.844	−103.688
*T*_0_				288.7
*T_m_/*K = *T*_0_ + ∑ *n_i_*∆*T_m,i_*				282.014
**GCM2**				
**Type**	**Group, *i***	***n_i_***	**∆*T_m,i_/K***	***n_i_*∆*T_m,i_/K***
**Cation**	N^+^	1	7.8678	7.8678
	CH_2_R_2_	2	1.6214	3.2428
	CH_3_X	1	12.4075	12.4075
	CH_2_RX	1	6.5888	6.5888
	R-CH-X	1	−45.1417	−45.1417
	R-CH..X	1	18.6936	18.6936
	H^a^ attached to C° (sp3) no X attached to next C	5	−1.3355	−6.6775
**Anion**	Sulfones (SO_2_)	2	−35.6122	−71.2244
	Total number of Ns, Os, and Fs in the molecule, excluding N with a formal positive charge, higher oxidation states, and pyrrolyl form of N	11	8.2032	90.2352
	CX_4_	2	−30.3021	−60.6042
	F^a^ attached to C^2^ (sp2)-C^4^ (sp2)/ C^1^ (sp)/C^4^ (sp3)/X	6	0.7619	4.5714
	R-SO_2_-R	2	22.9432	45.8864
*T*_0_				264.29
*T_m_/*K = *T*_0_ + ∑ *n_i_*∆*T_m,i_*				270.136

## Data Availability

The data presented in this study are available in the Supplementary Materials.

## Supplementary Materials

The Excel database of IL melting points is available online.

## Abbreviations

AARD	Average Absolute Relative Deviation
BETI	Bis((perfluoroethyl)sulfonyl)imide
CAMD	Computer Aided Molecular Design
DSC	Differential Scanning Calorimetry
GCM	Group Contribution Method
IL	Ionic Liquid
MAE	Mean Absolute Error
NTf_2_	Bis((trifluoromethyl)sulfonyl)imide
OTf	Trifluoromethanesulfonate
QSPR	Quantitative Structure Property Relationship
RNN	Recurrent Neural Network
SGD	Stochastic Gradient Descent

## References

[1] Welton T. (2018). Ionic liquids: A brief history. Biophys. Rev..

[2] Krossing I., Slattery J.M., Daguenet C., Dyson P.J., Oleinikova A., Weingärtner H. (2006). Why Are Ionic Liquids Liquid? A Simple Explanation Based on Lattice and Solvation Energies. J. Am. Chem. Soc..

[3] Greer A.J., Jacquemin J., Hardacre C. (2020). Industrial Applications of Ionic Liquids. Molecules.

[4] Ozokwelu D., Zhang S., Okafor O.C., Cheng W., Litombe N., Ozokwelu D., Zhang S., Okafor O.C., Cheng W., Litombe N. (2017). Chapter 5—Separation Science and Technology. Novel Catalytic and Separation Processes Based on Ionic Liquids.

[5] Chen H., He Y., Zhu J., Alias H., Ding Y., Nancarrow P., Hardacre C., Rooney D., Tan C. (2008). Rheological and heat transfer behaviour of the ionic liquid, [C4mim][NTf2]. Int. J. Heat Fluid Flow.

[6] Bendová M., Čanji M., Wagner Z., Bogdanov M.G. (2019). Ionic Liquids as Thermal Energy Storage Materials: On the Importance of Reliable Data Analysis in Assessing Thermodynamic Data. J. Solut. Chem..

[7] Nancarrow P., Al-Othman A., Mital D.K., Döpking S. (2021). Comprehensive analysis and correlation of ionic liquid conductivity data for energy applications. Energy.

[8] Mohammed H., Al-Othman A., Nancarrow P., Elsayed Y., Tawalbeh M. (2021). Enhanced proton conduction in zirconium phosphate/ionic liquids materials for high-temperature fuel cells. Int. J. Hydrogen Energy.

[9] Somers A.E., Howlett P.C., MacFarlane D.R., Forsyth M. (2013). A Review of Ionic Liquid Lubricants. Lubricants.

[10] Zhou Y., Qu J. (2017). Ionic Liquids as Lubricant Additives: A Review. Acs Appl. Mater. Interfaces.

[11] Nancarrow P., Mohammed H. (2017). Ionic Liquids in Space Technology—Current and Future Trends. ChemBioEng Rev..

[12] Schubert T.J.S., Shiflett M.B. (2020). Commercial Production of Ionic Liquids. Commercial Applications of Ionic Liquids.

[13] Ionic Liquids. https://chemicals.basf.com/global/en/Intermediates/Product_groups/Ionic_liquids.html.

[14] Zhu J., Bai L., Chen B., Fei W. (2009). Thermodynamical properties of phase change materials based on ionic liquids. Chem. Eng. J..

[15] Holbrey J.D., Seddon K.R. (1999). Ionic liquids. Clean Prod. Process..

[16] Niedermeyer H., Hallett J.P., Villar-Garcia I.J., Hunt P.A., Welton T. (2012). Mixtures of ionic liquids. Chem. Soc. Rev..

[17] Gani R. (2019). Group contribution-based property estimation methods: Advances and perspectives. Curr. Opin. Chem. Eng..

[18] Karelson M., Lobanov V.S., Katritzky A.R. (1996). Quantum-Chemical Descriptors in QSAR/QSPR Studies. Chem. Rev..

[19] Katritzky A.R., Lomaka A., Petrukhin R., Jain R., Karelson M., Visser A.E., Rogers R.D. (2002). QSPR Correlation of the Melting Point for Pyridinium Bromides, Potential Ionic Liquids. J. Chem. Inf. Comput. Sci..

[20] Lazzús J.A. (2012). A group contribution method to predict the melting point of ionic liquids. Fluid Phase Equilibria.

[21] Gharagheizi F., Ilani-Kashkouli P., Mohammadi A.H. (2012). Computation of normal melting temperature of ionic liquids using a group contribution method. Fluid Phase Equilibria.

[22] Gardas R.L., Coutinho J.A.P. (2008). Extension of the Ye and Shreeve group contribution method for density estimation of ionic liquids in a wide range of temperatures and pressures. Fluid Phase Equilibria.

[23] Jacquemin J., Ge R., Nancarrow P., Rooney D.W., Costa Gomes M.F., Pádua A.A.H., Hardacre C. (2008). Prediction of Ionic Liquid Properties. I. Volumetric Properties as a Function of Temperature at 0.1 MPa. J. Chem. Eng. Data.

[24] Jacquemin J., Nancarrow P., Rooney D.W., Costa Gomes M.F., Husson P., Majer V., Pádua A.A.H., Hardacre C. (2008). Prediction of Ionic Liquid Properties. II. Volumetric Properties as a Function of Temperature and Pressure. J. Chem. Eng. Data.

[25] Paduszyński K., Domańska U. (2012). A New Group Contribution Method For Prediction of Density of Pure Ionic Liquids over a Wide Range of Temperature and Pressure. Ind. Eng. Chem. Res..

[26] Evangelista N.S., do Carmo F.R., de Santiago-Aguiar R.S., de Sant’Ana H.B. (2014). Development of a new group contribution method based on GCVOL Model for the estimation of pure ionic liquid density over a wide range of temperature and pressure. Ind. Eng. Chem. Res..

[27] Součková M., Klomfar J., Pátek J. (2017). Group contribution and parachor analysis of experimental data on density and surface tension for members of the homologous series of 1-C -3-methylimidazolium chlorides. Fluid Phase Equilibria.

[28] Gardas R.L., Coutinho J.A.P. (2008). A group contribution method for viscosity estimation of ionic liquids. Fluid Phase Equilibria.

[29] Gharagheizi F., Ilani-Kashkouli P., Mohammadi A.H., Ramjugernath D., Richon D. (2012). Development of a group contribution method for determination of viscosity of ionic liquids at atmospheric pressure. Chem. Eng. Sci..

[30] Fatehi M.-R., Raeissi S., Mowla D. (2014). Estimation of viscosity of binary mixtures of ionic liquids and solvents using an artificial neural network based on the structure groups of the ionic liquid. Fluid Phase Equilibria.

[31] Lazzús J.A., Pulgar-Villarroel G. (2015). A group contribution method to estimate the viscosity of ionic liquids at different temperatures. J. Mol. Liq..

[32] Gardas R.L., Coutinho J.A.P. (2008). A group contribution method for heat capacity estimation of ionic liquids. Ind. Eng. Chem. Res..

[33] Valderrama J.O., Toro A., Rojas R.E. (2011). Prediction of the heat capacity of ionic liquids using the mass connectivity index and a group contribution method. J. Chem. Thermodyn..

[34] Sattari M., Gharagheizi F., Ilani-Kashkouli P., Mohammadi A.H., Ramjugernath D. (2014). Development of a group contribution method for the estimation of heat capacities of ionic liquids. J. Therm. Anal. Calorim.

[35] Nancarrow P., Lewis M., AbouChacra L. (2015). Group Contribution Methods for Estimation of Ionic Liquid Heat Capacities: Critical Evaluation and Extension. Chem. Eng. Technol..

[36] Ge R., Hardacre C., Jacquemin J., Nancarrow P., Rooney D.W. (2008). Heat Capacities of Ionic Liquids as a Function of Temperature at 0.1 MPa. Measurement and Prediction. J. Chem. Eng. Data.

[37] Lazzús J.A. (2015). A group contribution method to predict the thermal conductivity λ(T,P) of ionic liquids. Fluid Phase Equilibria.

[38] Atashrouz S., Mozaffarian M., Pazuki G. (2015). Modeling the Thermal Conductivity of Ionic Liquids and Ionanofluids Based on a Group Method of Data Handling and Modified Maxwell Model. Ind. Eng. Chem. Res..

[39] Wu K.-J., Zhao C.-X., He C.-H. (2013). Development of a group contribution method for determination of thermal conductivity of ionic liquids. Fluid Phase Equilibria.

[40] Joback K.G., Reid R.C. (1987). Estimation of Pure-Component Properties from Group-Contributions. Chem. Eng. Commun..

[41] Lydersen A.L., Engineering Experiment Station (1955). Estimation of Critical Properties of Organic Compounds by the Method of Group Contibutions.

[42] Huo Y., Xia S., Zhang Y., Ma P. (2009). Group Contribution Method for Predicting Melting Points of Imidazolium and Benzimidazolium Ionic Liquids. Ind. Eng. Chem. Res..

[43] Aguirre C.L., Cisternas L.A., Valderrama J.O. (2012). Melting-Point Estimation of Ionic Liquids by a Group Contribution Method. Int. J..

[44] Chen Y., Kontogeorgis G.M., Woodley J.M. (2019). Group Contribution Based Estimation Method for Properties of Ionic Liquids. Ind. Eng. Chem. Res..

[45] Katritzky A.R., Jain R., Lomaka A., Petrukhin R., Karelson M., Visser A.E., Rogers R.D. (2002). Correlation of the melting points of potential ionic liquids (imidazolium bromides and benzimidazolium bromides) using the CODESSA program. J. Chem. Inf. Comput. Sci..

[46] Trohalaki S., Pachter R. (2005). Prediction of Melting Points for Ionic Liquids. QSAR Comb. Sci..

[47] Farahani N., Gharagheizi F., Mirkhani S.A., Tumba K. (2012). Ionic liquids: Prediction of melting point by molecular-based model. Thermochim. Acta.

[48] Bini R., Chiappe C., Duce C., Micheli A., Solaro R., Starita A., Tiné M.R. (2008). Ionic liquids: Prediction of their melting points by a recursive neural network model. Green Chem..

[49] Carrera G., Aires-de-Sousa J. (2005). Estimation of melting points of pyridinium bromide ionic liquids with decision trees and neural networks. Green Chem..

[50] Zhang S., Sun N., He X., Lu X., Zhang X. (2006). Physical Properties of Ionic Liquids: Database and Evaluation. J. Phys. Chem. Ref. Data.

[51] Eike D.M., Brennecke J.F., Maginn E.J. (2003). Predicting melting points of quaternary ammonium ionic liquids. Green Chem..

[52] Varnek A., Kireeva N., Tetko I.V., Baskin I.I., Solov’ev V.P. (2007). Exhaustive QSPR Studies of a Large Diverse Set of Ionic Liquids:  How Accurately Can We Predict Melting Points?. J. Chem. Inf. Model..

[53] López-Martin I., Burello E., Davey P.N., Seddon K.R., Rothenberg G. (2007). Anion and cation effects on imidazolium salt melting points: A descriptor modelling study. Chemphyschem.

[54] Yan C., Han M., Wan H., Guan G. (2010). QSAR correlation of the melting points for imidazolium bromides and imidazolium chlorides ionic liquids. Fluid Phase Equilibria.

[55] Valderrama J.O., Campusano R.A. (2016). Melting properties of molten salts and ionic liquids. Chemical homology, correlation, and prediction. Comptes Rendus Chim..

[56] Gill P., Moghadam T.T., Ranjbar B. (2010). Differential scanning calorimetry techniques: Applications in biology and nanoscience. J. Biomol Tech..

[57] Kočí V., Fořt J., Maděra J., Scheinherrová L., Trník A., Černý R. (2020). Correction of errors in DSC measurements using detailed modeling of thermal phenomena in calorimeter-sample system. IEEE Trans. Instrum. Meas..

[58] Preiss U.P., Beichel W., Erle A.M.T., Paulechka Y.U., Krossing I. (2011). Is universal, simple melting point prediction possible?. ChemPhysChem.

[59] Yamamoto H. (2006). Structure Properties Relationship of Ionic Liquid. J. Comput. Aided Chem..

[60] Zhang Y., Maginn E.J. (2014). Molecular dynamics study of the effect of alkyl chain length on melting points of [CnMIM][PF6] ionic liquids. Phys. Chem. Chem. Phys..

[61] (2020). NIST Ionic Liquids Database–ILThermo (v.2.0). https://ilthermo.boulder.nist.gov/.

[62] Zhang S., Zhou Q., Lu X., Song Y., Wang X. (2016). Introduction to properties of ionic liquid mixtures. Physicochemical Properties of Ionic Liquid Mixtures.

[63] Krolikowska M. (2014). (Solid + liquid) and (liquid + liquid) phase equilibria of (IL + water) binary systems. The influence of the ionic liquid structure on mutual solubility. Fluid Phase Equilibria.

[64] Yoshida Y., Muroi K., Otsuka A., Saito G., Takahashi M., Yoko T. (2004). 1-Ethyl-3-methylimidazolium based ionic liquids containing cyano groups: Synthesis, characterization, and crystal structure. Inorg. Chem..

[65] Wasserscheid P., van Hal R., Bösmann A. (2002). 1-n-Butyl-3-methylimidazolium ([bmim]) octylsulfate—an even ‘greener’ionic liquid. Green Chem..

[66] Domańska U., Bogel-Łukasik E., Bogel-Łukasik R. (2003). 1-Octanol/Water Partition Coefficients of 1Alkyl-3-methylimidazolium Chloride. Chem. A Eur. J..

[67] Couadou E., Jacquemin J., Galiano H., Hardacre C., Anouti M. (2013). A comparative study on the thermophysical properties for two bis [(trifluoromethyl) sulfonyl] imide-based ionic liquids containing the trimethyl-sulfonium or the trimethyl-ammonium cation in molecular solvents. J. Phys. Chem. B.

[68] Zhou Z.-B., Matsumoto H., Tatsumi K. (2004). A new class of hydrophobic ionic liquids: Trialkyl (2-methoxyethyl) ammonium perfluoroethyltrifluoroborate. Chem. Lett..

[69] Sun J., Macfarlane D.R., Forsyth M. (2003). A new family of ionic liquids based on the 1-alkyl-2-methyl pyrrolinium cation. Electrochim. Acta.

[70] Sakal S.A., Lu Y., Jiang X., Shen C., Li C. (2014). A promising ionic liquid [BMIM][FeCl4] for the extractive separation of aromatic and aliphatic hydrocarbons. J. Chem. Eng. Data.

[71] Wlazło M., Marciniak A., Zawadzki M., Dudkiewicz B. (2015). Activity coefficients at infinite dilution and physicochemical properties for organic solutes and water in the ionic liquid 4-(3-hydroxypropyl)-4-methylmorpholinium bis (trifluoromethylsulfonyl)-amide. J. Chem. Thermodyn..

[72] Wilkes J.S., Zaworotko M.J. (1992). Air and water stable 1-ethyl-3-methylimidazolium based ionic liquids. J. Chem. Soc. Chem. Commun..

[73] Mikkola J.-P., Virtanen P., Sjöholm R. (2006). Aliquat 336^®^—a versatile and affordable cation source for an entirely new family of hydrophobic ionic liquids. Green Chem..

[74] Cooper E.I., Angell C.A. (1986). Ambient temperature plastic crystal fast ion conductors (PLICFICS). Solid State Ion..

[75] Melo C.I., Bogel-Łukasik R., da Ponte M.N., Bogel-Łukasik E. (2013). Ammonium ionic liquids as green solvents for drugs. Fluid Phase Equilibria.

[76] Takaizumi K., Wakabayashi T. (1980). Apparent molal volumes of phosphonium halides at 15, 25 and 35 °C. J. Solut. Chem..

[77] De Roche J., Gordon C.M., Imrie C.T., Ingram M.D., Kennedy A.R., Lo Celso F., Triolo A. (2003). Application of complementary experimental techniques to characterization of the phase behavior of [C16mim][PF6] and [C14mim][PF6]. Chem. Mater..

[78] Marszalek M., Fei Z., Zhu D.-R., Scopelliti R., Dyson P.J., Zakeeruddin S.M., Grätzel M. (2011). Application of Ionic Liquids Containing Tricyanomethanide [C(CN)3]− or Tetracyanoborate [B(CN)4]− Anions in Dye-Sensitized Solar Cells. Inorg. Chem..

[79] Vataščin E., Dohnal V. (2018). Aqueous solutions of [EMIM] 1, 1, 2, 2-tetrafluoroethanesulfonate and [EMIM] trifluoromethanesulfonate: A thermodynamic study. J. Chem. Thermodyn..

[80] Verevkin S.P., Zaitsau D.H., Emel’yanenko V.N., Ralys R.V., Schick C., Geppert-Rybczyńska M., Jayaraman S., Maginn E.J. (2012). Benchmark values: Thermochemistry of the ionic liquid [C4Py][Cl]. Aust. J. Chem..

[81] Murugesan S., Wiencek J.M., Ren R.X., Linhardt R.J. (2006). Benzoate-based room temperature ionic liquids—thermal properties and glycosaminoglycan dissolution. Carbohydr. Polym..

[82] Gathergood N., Garcia M.T., Scammells P.J. (2004). Biodegradable ionic liquids: Part I. Concept, preliminary targets and evaluation. Green Chem..

[83] Singh R.P., Shreeve J.M. (2003). Bridged Tetraquaternary Salts from N,N′-Polyfluoroalkyl-4,4′-bipyridine. Inorg. Chem..

[84] Brünig T., Krekić K., Bruhn C., Pietschnig R. (2016). Calorimetric studies and structural aspects of ionic liquids in designing sorption materials for thermal energy storage. Chemistry.

[85] Abranches D.O., Silva L.P., Martins M.A.R., Fernandez L., Pinho S.P., Coutinho J.A.P. (2019). Can cholinium chloride form eutectic solvents with organic chloride-based salts?. Fluid Phase Equilibria.

[86] Shim J.-J., Kim D., Ra C.-S. (2006). Carboxylation of styrene oxide catalyzed by quaternary onium salts under solvent-free conditions. Bull. Korean Chem. Soc..

[87] Kabachnik M.I., Zakharov L.S., Kudryavtsev I.Y. (1989). Catalytic phosphorylation of polyfluoroalkanols 13. Some ammonium and phosphonium salts as phosphorylation catalysts. Russ. Chem Bull..

[88] Sheldon R. (2001). Catalytic reactions in ionic liquids. Chem. Commun..

[89] Huddleston J.G., Visser A.E., Reichert W.M., Willauer H.D., Broker G.A., Rogers R.D. (2001). Characterization and comparison of hydrophilic and hydrophobic room temperature ionic liquids incorporating the imidazolium cation. Green Chem..

[90] Morita T., Nitta A., Nishikawa K., Westh P., Koga Y. (2014). Characterization of BF4− in terms of its effect on water by the 1-propanol probing methodology. J. Mol. Liq..

[91] Visser A.E., Reichert W.M., Swatloski R.P., Willauer H.D., Huddleston J.G., Rogers R.D. (2002). Characterization of Hydrophilic and Hydrophobic Ionic Liquids: Alternatives to Volatile Organic Compounds for Liquid-Liquid Separations. Ionic Liquids.

[92] Appetecchi G.B., Montanino M., Carewska M., Moreno M., Alessandrini F., Passerini S. (2011). Chemical–physical properties of bis (perfluoroalkylsulfonyl) imide-based ionic liquids. Electrochim. Acta.

[93] Nockemann P., Thijs B., Driesen K., Janssen C.R., Van Hecke K., Van Meervelt L., Kossmann S., Kirchner B., Binnemans K. (2007). Choline saccharinate and choline acesulfamate: Ionic liquids with low toxicities. J. Phys. Chem. B.

[94] Shimizu Y., Ohte Y., Yamamura Y., Tsuzuki S., Saito K. (2012). Comparative study of imidazolium-and pyrrolidinium-based ionic liquids: Thermodynamic properties. J. Phys. Chem. B.

[95] Yasuda T., Kinoshita H., Miran M.S., Tsuzuki S., Watanabe M. (2013). Comparative study on physicochemical properties of protic ionic liquids based on allylammonium and propylammonium cations. J. Chem. Eng. Data.

[96] Kulkarni P.S., Branco L.C., Crespo J.G., Nunes M.C., Raymundo A., Afonso C.A. (2007). Comparison of physicochemical properties of new ionic liquids based on imidazolium, quaternary ammonium, and guanidinium cations. Chem. A Eur. J..

[97] Aranowski R., Cichowska-Kopczyńska I., Dębski B., Jasiński P. (2016). Conductivity and viscosity changes of imidazolium ionic liquids induced by H_2_O and CO_2_. J. Mol. Liq..

[98] Holbrey J.D., Reichert W.M., Nieuwenhuyzen M., Johnson S., Seddon K.R., Rogers R.D. (2003). Crystal polymorphism in 1-butyl-3-methylimidazolium halides: Supporting ionic liquid formation by inhibition of crystallization. Chem. Commun..

[99] Zhou Z.-B., Matsumoto H., Tatsumi K. (2006). Cyclic quaternary ammonium ionic liquids with perfluoroalkyltrifluoroborates: Synthesis, characterization, and properties. Chem. A Eur. J..

[100] Yang D., Zhang S., Sun X., Jiang D., Dai S. (2019). Deep eutectic solvents formed by quaternary ammonium salts and aprotic organic compound succinonitrile. J. Mol. Liq..

[101] Liu Q.-S., Yang M., Yan P.-F., Liu X.-M., Tan Z.-C., Welz-Biermann U. (2010). Density and surface tension of ionic liquids [C n py][NTf2] (n = 2, 4, 5). J. Chem. Eng. Data.

[102] Królikowska M., Lipiński P., Maik D. (2014). Density, viscosity and phase equilibria study of {ethylsulfate-based ionic liquid+water} binary systems as a function of temperature and composition. Thermochim. Acta.

[103] Larsen A.S., Holbrey J.D., Tham F.S., Reed C.A. (2000). Designing Ionic Liquids:  Imidazolium Melts with Inert Carborane Anions. J. Am. Chem. Soc..

[104] Wachter P., Schreiner C., Schweiger H.-G., Gores H.J. (2010). Determination of phase transition points of ionic liquids by combination of thermal analysis and conductivity measurements at very low heating and cooling rates. J. Chem. Thermodyn..

[105] Wilkes J.S., Levisky J.A., Wilson R.A., Hussey C.L. (1982). Dialkylimidazolium chloroaluminate melts: A new class of room-temperature ionic liquids for electrochemistry, spectroscopy and synthesis. Inorg. Chem..

[106] Zhao D., Fei Z., Ohlin C.A., Laurenczy G., Dyson P.J. (2004). Dual-functionalised ionic liquids: Synthesis and characterisation of imidazolium salts with a nitrile-functionalised anion. Chem. Commun..

[107] Mukherjee P., Crank J.A., Sharma P.S., Wijeratne A.B., Adhikary R., Bose S., Armstrong D.W., Petrich J.W. (2008). Dynamic solvation in phosphonium ionic liquids: Comparison of bulk and micellar systems and considerations for the construction of the solvation correlation function, C (t). J. Phys. Chem. B.

[108] Xue L., Gurung E., Tamas G., Koh Y.P., Shadeck M., Simon S.L., Maroncelli M., Quitevis E.L. (2016). Effect of alkyl chain branching on physicochemical properties of imidazolium-based ionic liquids. J. Chem. Eng. Data.

[109] Shen C., Li X., Lu Y., Li C. (2011). Effect of ionic liquid 1-methylimidazolium chloride on the vapour liquid equilibrium of water, methanol, ethanol, and {water+ ethanol} mixture. J. Chem. Thermodyn..

[110] Khan A.S., Man Z., Bustam M.A., Gonfa G., Chong F.K., Ullah Z., Nasrullah A., Sarwono A., Ahmad P., Muhammad N. (2017). Effect of structural variations on the thermophysical properties of protic ionic liquids: Insights from experimental and computational studies. J. Chem. Eng. Data.

[111] Pereiro A.B., Veiga H.I., Esperança J.M., Rodríguez A. (2009). Effect of temperature on the physical properties of two ionic liquids. J. Chem. Thermodyn..

[112] Shimizu Y., Ohte Y., Yamamura Y., Saito K. (2007). Effects of thermal history on thermal anomaly in solid of ionic liquid compound,[C4mim][Tf2N]. Chem. Lett..

[113] Wachter P., Schweiger H.-G., Wudy F., Gores H.J. (2008). Efficient determination of crystallisation and melting points at low cooling and heating rates with novel computer controlled equipment. J. Chem. Thermodyn..

[114] Makino T., Kanakubo M., Umecky T., Suzuki A. (2013). Electrical Conductivities, Viscosities, and Densities of N-Acetoxyethyl-N, N-dimethyl-N-ethylammonium and N, N-Dimethyl-N-ethyl-N-methoxyethoxyethylammonium Bis (trifluoromethanesulfonyl) amide and Their Nonfunctionalized Analogues. J. Chem. Eng. Data.

[115] McEwen A.B., Ngo H.L., LeCompte K., Goldman J.L. (1999). Electrochemical properties of imidazolium salt electrolytes for electrochemical capacitor applications. J. Electrochem. Soc..

[116] Xue H., Arritt S.W., Twamley B., Shreeve J.M. (2004). Energetic salts from N-aminoazoles. Inorg. Chem..

[117] Suarez P.A.Z., Selbach V.M., Dullius J.E.L., Einloft S., Piatnicki C.M.S., Azambuja D.S., de Souza R.F., Dupont J. (1997). Enlarged electrochemical window in dialkyl-imidazolium cation based room-temperature air and water-stable molten salts. Electrochim. Acta.

[118] Stolarska O., Rodriguez H., Smiglak M. (2016). Eutectic mixtures of pyrrolidinium-based ionic liquids. Fluid Phase Equilibria.

[119] Zheng L., Pan Y., Ji H.-X., Ma X.-X., Xing N.-N., Guan W. (2016). Evaluation of the Walden Product of Ionic Liquids Using Experiments and a New Theory: An Ion Exchange Transition Model. Acta Phys. Chim. Sin..

[120] Zhang D., Chen J., Liang Y., Zhou H. (2005). Facile synthesis of novel ionic liquids containing dithiocarbamate. Synth. Commun..

[121] Vieira N.S., Luís A., Reis P.M., Carvalho P.J., Lopes-Da-Silva J.A., Esperança J., Araújo J.M.M., Rebelo L.P.N., Freire M.G., Pereiro A.B. (2016). Fluorination effects on the thermodynamic, thermophysical and surface properties of ionic liquids. J. Chem. Thermodyn..

[122] Nishi N., Kawakami T., Shigematsu F., Yamamoto M., Kakiuchi T. (2006). Fluorine-free and hydrophobic room-temperature ionic liquids, tetraalkylammonium bis (2-ethylhexyl) sulfosuccinates, and their ionic liquid–water two-phase properties. Green Chem..

[123] Coker T.G., Ambrose J., Janz G.J. (1970). Fusion properties of some ionic quaternary ammonium compounds. J. Am. Chem. Soc..

[124] Moura Ramos J.J., Afonso C.A.M., Branco L.C. (2003). Glass transition relaxation and fragility in two room temperature ionic liquids. J. Therm. Anal. Calorim..

[125] Bhatt A.I., May I., Volkovich V.A., Hetherington M.E., Lewin B., Thied R.C., Ertok N. (2002). Group 15 quaternary alkyl bistriflimides: Ionic liquids with potential application in electropositive metal deposition and as supporting electrolytes. J. Chem. Soc., Dalton Trans..

[126] Hughes T.J., Syed T., Graham B.F., Marsh K.N., May E.F. (2011). Heat capacities and low temperature thermal transitions of 1-hexyl and 1-octyl-3-methylimidazolium bis (trifluoromethylsulfonyl) amide. J. Chem. Eng. Data.

[127] Zimmer M.F., Baroody E.E., Carpenter G.A., Robb R.A. (1968). Heat of formation of hydroxylammonium perchlorate by combustion calorimetry. J. Chem. Eng. Data.

[128] McFarlane D.R., Sun J., Golding J., Meakin P., Forsyth M. (2000). High conductivity molten salts based on the imide ion. Electrochim. Acta.

[129] Anderson J.L., Armstrong D.W. (2003). High-stability ionic liquids. A new class of stationary phases for gas chromatography. Anal. Chem..

[130] Noda A., Watanabe M. (2000). Highly conductive polymer electrolytes prepared by in situ polymerization of vinyl monomers in room temperature molten salts. Electrochim. Acta.

[131] Matsumoto H., Yanagida M., Tanimoto K., Nomura M., Kitagawa Y., Miyazaki Y. (2000). Highly conductive room temperature molten salts based on small trimethylalkylammonium cations and bis (trifluoromethylsulfonyl) imide. Chem. Lett..

[132] Zeng S., Wang J., Bai L., Wang B., Gao H., Shang D., Zhang X., Zhang S. (2015). Highly selective capture of CO2 by ether-functionalized pyridinium ionic liquids with low viscosity. Energy Fuels.

[133] Tokuda H., Tsuzuki S., Susan M.A.B.H., Hayamizu K., Watanabe M. (2006). How ionic are room-temperature ionic liquids? An indicator of the physicochemical properties. J. Phys. Chem. B.

[134] Bonhote P., Dias A.-P., Papageorgiou N., Kalyanasundaram K., Grätzel M. (1996). Hydrophobic, highly conductive ambient-temperature molten salts. Inorg. Chem..

[135] Kato H., Miki K., Mukai T., Nishikawa K., Koga Y. (2009). Hydrophobicity/hydrophilicity of 1-butyl-2, 3-dimethyl and 1-ethyl-3-methylimodazolium ions: Toward characterization of room temperature ionic liquids. J. Phys. Chem. B.

[136] Sun J., Zhang S., Cheng W., Ren J. (2008). Hydroxyl-functionalized ionic liquid: A novel efficient catalyst for chemical fixation of CO_2_ to cyclic carbonate. Tetrahedron Lett..

[137] Emel’yanenko V.N., Verevkin S.P., Heintz A., Voss K., Schulz A. (2009). Imidazolium-based ionic liquids. 1-Methyl imidazolium nitrate: Thermochemical measurements and Ab initio calculations. J. Phys. Chem. B.

[138] Matsumoto H., Yanagida M., Tanimoto K., Kojima T., Tamiya Y., Miyazaki Y. (1999). Improvement of Ionic Conductivity of Room Temperature Molten Salt Based on Quaternary Ammonium Cation and Imide Anion. Proc. Vol..

[139] Meyer K.L., Marasco C.J., Morris-Natschke S.L., Ishaq K.S., Piantadosi C., Kucera L.S. (1991). In vitro evaluation of phosphocholine and quaternary ammonium containing lipids as novel anti-HIV agents. J. Med. Chem..

[140] Govinda V., Reddy P.M., Attri P., Venkatesu P., Venkateswarlu P. (2013). Influence of anion on thermophysical properties of ionic liquids with polar solvent. J. Chem. Thermodyn..

[141] Domańska U., Morawski P. (2007). Influence of high pressure on solubility of ionic liquids: Experimental data and correlation. Green Chem..

[142] Rauber D., Heib F., Schmitt M., Hempelmann R. (2016). Influence of perfluoroalkyl-chains on the surface properties of 1-methylimidazolium bis (trifluoromethanesulfonyl) imide ionic liquids. J. Mol. Liq..

[143] Chun S., Dzyuba S.V., Bartsch R.A. (2001). Influence of structural variation in room-temperature ionic liquids on the selectivity and efficiency of competitive alkali metal salt extraction by a crown ether. Anal. Chem..

[144] Dzyuba S.V., Bartsch R.A. (2002). Influence of structural variations in 1-alkyl (aralkyl)-3-methylimidazolium hexafluorophosphates and bis (trifluoromethylsulfonyl) imides on physical properties of the ionic liquids. ChemPhysChem.

[145] Hofmann A., Migeot M., Hanemann T. (2016). Investigation of binary mixtures containing 1-ethyl-3-methylimidazolium bis (trifluoromethanesulfonyl) azanide and ethylene carbonate. J. Chem. Eng. Data.

[146] Ohno H., Yoshizawa M. (2002). Ion conductive characteristics of ionic liquids prepared by neutralization of alkylimidazoles. Solid State Ion..

[147] Gordon C.M., Holbrey J.D., Kennedy A.R., Seddon K.R. (1998). Ionic liquid crystals: Hexafluorophosphate salts. J. Mater. Chem..

[148] Yang C., Sun Q., Qiao J., Li Y. (2003). Ionic liquid doped polymer light-emitting electrochemical cells. J. Phys. Chem. B.

[149] Annat G., Forsyth M., MacFarlane D.R. (2012). Ionic Liquid Mixtures Variations in Physical Properties and Their Origins in Molecular Structure. J. Phys. Chem. B.

[150] Kim K.-S., Choi S., Demberelnyamba D., Lee H., Oh J., Lee B.-B., Mun S.-J. (2004). Ionic liquids based on N-alkyl-N-methylmorpholinium salts as potential electrolytes. Chem. Commun..

[151] Vieira M.O., Monteiro W.F., Ligabue R., Seferin M., Chaban V.V., Andreeva N.A., do Nascimento J.F., Einloft S. (2017). Ionic liquids composed of linear amphiphilic anions: Synthesis, physicochemical characterization, hydrophilicity and interaction with carbon dioxide. J. Mol. Liq..

[152] Lee J.S., Quan N.D., Hwang J.M., Bae J.Y., Kim H., Cho B.W., Kim H.S., Lee H. (2006). Ionic liquids containing an ester group as potential electrolytes. Electrochem. Commun..

[153] De Gaetano Y., Mohamadou A., Boudesocque S., Hubert J., Plantier-Royon R., Dupont L. (2015). Ionic liquids derived from esters of Glycine Betaine: Synthesis and characterization. J. Mol. Liq..

[154] Berthod A., Ruiz-Angel M.J., Carda-Broch S. (2008). Ionic liquids in separation techniques. J. Chromatogr. A.

[155] Xu W., Wang L.-M., Nieman R.A., Angell C.A. (2003). Ionic liquids of chelated orthoborates as model ionic glassformers. J. Phys. Chem. B.

[156] Olivier-Bourbigou H., Magna L. (2002). Ionic liquids: Perspectives for organic and catalytic reactions. J. Mol. Catal. A Chem..

[157] Zhang S., Lu X., Zhou Q., Li X., Zhang X., Li S. (2009). Ionic Liquids: Physicochemical Properties.

[158] Emel’yanenko V.N., Verevkin S.P., Heintz A., Schick C. (2008). Ionic liquids. Combination of combustion calorimetry with high-level quantum chemical calculations for deriving vaporization enthalpies. J. Phys. Chem. B.

[159] Morais A.R., da Costa Lopes A.M., Bogel-Łukasik E., Bogel-Łukasik R. (2013). Ionic liquids’ cation and anion influence on aromatic amine solubility. Ind. Eng. Chem. Res..

[160] Ma X.-X., Wei J., Guan W., Pan Y., Zheng L., Wu Y., Yang J.-Z. (2015). Ionic parachor and its application to pyridinium-based ionic liquids of {[Cnpy][DCA](n = 2, 3, 4, 5, 6)}. J. Chem. Thermodyn..

[161] Shimizu Y., Ohte Y., Yamamura Y., Saito K. (2009). Is the liquid or the solid phase responsible for the low melting points of ionic liquids? Alkyl-chain-length dependence of thermodynamic properties of [Cnmim][Tf2N]. Chem. Phys. Lett..

[162] Kurzin A.V., Evdokimov A.N., Feofanova M.A., Baranova N.V. (2017). Isothermal Vapor–Liquid Equilibrium Data for the Toluene+ Methanol+ N-Butylpyridinium Bromide System. J. Chem. Eng. Data.

[163] Elias H., Strecker H. (1966). Kinetik des homogenen Isotopenaustausches zwischen Isopropylchlorid und Chlorid-Ionen in Dimethylformamid. Chem. Ber..

[164] Kissinger P., Heineman W.R. (2018). Laboratory Techniques in Electroanalytical Chemistry, Revised and Expanded.

[165] MacFarlane D.R., Pringle J.M., Johansson K.M., Forsyth S.A., Forsyth M. (2006). Lewis base ionic liquids. Chem. Commun..

[166] Schneider S., Hawkins T., Rosander M., Mills J., Brand A., Hudgens L., Warmoth G., Vij A. (2008). Liquid azide salts. Inorg. Chem..

[167] Le M.L.P., Tran N.A., Ngo H.P.K., Nguyen T.G. (2015). Liquid electrolytes based on ionic liquids for lithium-ion batteries. J. Solut. Chem..

[168] Villanueva M., Parajó J.J., Sánchez P.B., García J., Salgado J. (2015). Liquid range temperature of ionic liquids as potential working fluids for absorption heat pumps. J. Chem. Thermodyn..

[169] Parajó J.J., Villanueva M., Sánchez P.B., Salgado J. (2018). Liquid window of some biologically-active ionic liquids. J. Chem. Thermodyn..

[170] Castro M.C., Arce A., Soto A., Rodríguez H. (2016). Liquid-liquid equilibria of mutually immiscible ionic liquids with a common anion of basic character. J. Chem. Thermodyn..

[171] Domańska U., Pobudkowska A., Eckert F. (2006). Liquid–liquid equilibria in the binary systems (1,3-dimethylimidazolium, or 1-butyl-3-methylimidazolium methylsulfate+ hydrocarbons). Green Chem..

[172] Paduszyński K., Chiyen J., Ramjugernath D., Letcher T.M., Domańska U. (2011). Liquid–liquid phase equilibrium of (piperidinium-based ionic liquid+ an alcohol) binary systems and modelling with NRHB and PCP-SAFT. Fluid Phase Equilibria.

[173] Vila J., Fernández-Castro B., Rilo E., Carrete J., Domínguez-Pérez M., Rodríguez J.R., García M., Varela L.M., Cabeza O. (2012). Liquid–solid–liquid phase transition hysteresis loops in the ionic conductivity of ten imidazolium-based ionic liquids. Fluid Phase Equilibria.

[174] Sahandzhieva K., Naydenov D., Pérez-Salado Kamps Á., Bart H.-J., Maurer G. (2010). Liquid−Liquid Equilibrium in Systems with an Ionic Liquid: Experimental Data for an Example of the Biphasic Acid Scavenging Utilizing Ionic Liquids Process. J. Chem. Eng. Data.

[175] Trindade C.A., Visak Z.P., Bogel-Łukasik R., Bogel-Łukasik E., da Ponte M.N. (2010). Liquid-liquid equilibrium of mixtures of imidazolium-based ionic liquids with propanediols or glycerol. Ind. Eng. Chem. Res..

[176] Pernak J., Smiglak M., Griffin S.T., Hough W.L., Wilson T.B., Pernak A., Zabielska-Matejuk J., Fojutowski A., Kita K., Rogers R.D. (2006). Long alkyl chain quaternary ammonium-based ionic liquids and potential applications. Green Chem..

[177] Kim J., Singh R.P., Shreeve J.M. (2004). Low melting inorganic salts of alkyl-, fluoroalkyl-, alkyl ether-, and fluoroalkyl ether-substituted oxazolidine and morpholine. Inorg. Chem..

[178] Mirzaei Y.R., Xue H., Shreeve J.M. (2004). Low melting N-4-functionalized-1-alkyl or polyfluoroalkyl-1,2,4-triazolium salts. Inorg. Chem..

[179] MacFarlane D.R., Golding J., Forsyth S., Forsyth M., Deacon G.B. (2001). Low viscosity ionic liquids based on organic salts of the dicyanamide anion. Chem. Commun..

[180] Zhou Z.-B., Matsumoto H., Tatsumi K. (2004). Low-melting, low-viscous, hydrophobic ionic liquids: 1-alkyl (alkyl ether)-3-methylimidazolium perfluoroalkyltrifluoroborate. Chem. A Eur. J..

[181] Zhou Z.-B., Matsumoto H., Tatsumi K. (2005). Low-melting, low-viscous, hydrophobic ionic liquids: Aliphatic quaternary ammonium salts with perfluoroalkyltrifluoroborates. Chem. A Eur. J..

[182] Fang D., Luo J., Xin-Li Z., Zu-Liang L. (2007). Mannich reaction in water using acidic ionic liquid as recoverable and reusable catalyst. Catal. Lett..

[183] Greaves T.L., Weerawardena A., Fong C., Drummond C.J. (2007). Many protic ionic liquids mediate hydrocarbon-solvent interactions and promote amphiphile self-assembly. Langmuir.

[184] Pontes P.V., Crespo E.A., Martins M.A., Silva L.P., Neves C.M., Maximo G.J., Hubinger M.D., Batista E.A., Pinho S.P., Coutinho J.A. (2017). Measurement and PC-SAFT modeling of solid-liquid equilibrium of deep eutectic solvents of quaternary ammonium chlorides and carboxylic acids. Fluid Phase Equilibria.

[185] Domańska U., Bogel-Łukasik E. (2003). Measurements and Correlation of the (Solid + Liquid) Equilibria of [1-Decyl-3-methylimidazolium Chloride + Alcohols (C2−C12)]. Ind. Eng. Chem. Res..

[186] Nishikawa K., Wang S., Katayanagi H., Hayashi S., Hamaguchi H., Koga Y., Tozaki K. (2007). Melting and freezing behaviors of prototype ionic liquids, 1-butyl-3-methylimidazolium bromide and its chloride, studied by using a nano-watt differential scanning calorimeter. J. Phys. Chem. B.

[187] Weiss H., Mars J., Li H., Kircher G., Ivanova O., Feoktystov A., Soltwedel O., Bier M., Mezger M. (2017). Mesoscopic correlation functions in heterogeneous ionic liquids. J. Phys. Chem. B.

[188] Golding J., Forsyth S., Macfarlane D.R., Forsyth M., Deacon G.B. (2002). Methanesulfonate and p-toluenesulfonate salts of the N-methyl-N-alkylpyrrolidinium and quaternary ammonium cations: Novel low cost ionic liquids. Green Chem..

[189] Hill A.J., Huang J., Efthimiadis J., Meakin P., Forsyth M., MacFarlane D.R. (2002). Microstructural and molecular level characterisation of plastic crystal phases of pyrrolidinium trifluoromethanesulfonyl salts. Solid State Ion..

[190] Le L.T., Vo T.D., Ngo K.H., Okada S., Alloin F., Garg A., Le P.M. (2018). Mixing ionic liquids and ethylene carbonate as safe electrolytes for lithium-ion batteries. J. Mol. Liq..

[191] Hu H.-C., Soriano A.N., Leron R.B., Li M.-H. (2011). Molar heat capacity of four aqueous ionic liquid mixtures. Thermochim. Acta.

[192] Forsyth S.A., Fraser K.J., Howlett P.C., MacFarlane D.R., Forsyth M. (2006). N-methyl-N-alkylpyrrolidinium nonafluoro-1-butanesulfonate salts: Ionic liquid properties and plastic crystal behaviour. Green Chem..

[193] Sakaebe H., Matsumoto H. (2003). N-Methyl-N-propylpiperidinium bis (trifluoromethanesulfonyl) imide (PP13–TFSI)–novel electrolyte base for Li battery. Electrochem. Commun..

[194] Xue H., Gao Y., Twamley B., Shreeve J.M. (2005). New energetic salts based on nitrogen-containing heterocycles. Chem. Mater..

[195] Tao G., He L., Sun N., Kou Y. (2005). New generation ionic liquids: Cations derived from amino acids. Chem. Commun..

[196] Pernak J., Czepukowicz A., Poźniak R. (2001). New ionic liquids and their antielectrostatic properties. Ind. Eng. Chem. Res..

[197] Holbrey J.D., Turner M.B., Reichert W.M., Rogers R.D. (2003). New ionic liquids containing an appended hydroxyl functionality from the atom-efficient, one-pot reaction of 1-methylimidazole and acid with propylene oxide. Green Chem..

[198] Ignat’ev N.V., Welz-Biermann U., Kucheryna A., Bissky G., Willner H. (2005). New ionic liquids with tris (perfluoroalkyl) trifluorophosphate (FAP) anions. J. Fluor. Chem..

[199] Wasserscheid P., van Hal R., Bösmann A. (2002). New, Halogen-Free Ionic Liquids—Synthesis, Properties and Applications. ECS Proc. Vol..

[200] Zhao D., Fei Z., Geldbach T.J., Scopelliti R., Dyson P.J. (2004). Nitrile-functionalized pyridinium ionic liquids: Synthesis, characterization, and their application in carbon− carbon coupling reactions. J. Am. Chem. Soc..

[201] Xing H., Wang T., Zhou Z., Dai Y. (2005). Novel Brønsted-acidic ionic liquids for esterifications. Ind. Eng. Chem. Res..

[202] Wu B., Reddy R.G., Rogers R.D. Novel ionic liquid thermal storage for solar thermal electric power systems. Proceedings of the International Solar Energy Conference.

[203] Yagupolskii Y.L., Sokolenko T.M., Petko K.I., Yagupolskii L.M. (2005). Novel ionic liquids—Imidazolium salts with a difluoromethylene fragment directly bonded to the nitrogen atom. J. Fluor. Chem..

[204] Pereiro A.B., Pastoriza-Gallego M.J., Shimizu K., Marrucho I.M., Lopes J.N.C., Piñeiro M.M., Rebelo L.P.N. (2013). On the formation of a third, nanostructured domain in ionic liquids. J. Phys. Chem. B.

[205] Dong F., Jun L., Xinli Z., Zhiwen Y., Zuliang L. (2007). One-pot green procedure for Biginelli reaction catalyzed by novel task-specific room-temperature ionic liquids. J. Mol. Catal. A Chem..

[206] Fei Z., Zhao D., Scopelliti R., Dyson P.J. (2004). Organometallic complexes derived from alkyne-functionalized imidazolium salts. Organometallics.

[207] Lapidus A., Eliseev O., Bondarenko T., Stepin N. (2006). Palladium catalysed hydroxycarbonylation of 1-phenylethanol in molten salt media. J. Mol. Catal. A Chem..

[208] Henderson W.A., Passerini S. (2004). Phase behavior of ionic liquid− LiX mixtures: Pyrrolidinium cations and TFSI-anions. Chem. Mater..

[209] Štejfa V., Rohlíček J., Červinka C. (2020). Phase behaviour and heat capacities of selected 1-ethyl-3-methylimidazolium-based ionic liquids. J. Chem. Thermodyn..

[210] Domańska U., Królikowska M. (2011). Phase behaviour of 1-butyl-1-methylpyrrolidinium thiocyanate ionic liquid. Fluid Phase Equilibria.

[211] Chen H., Kwait D.C., Gönen Z.S., Weslowski B.T., Abdallah D.J., Weiss R.G. (2002). Phase characterization and properties of completely saturated quaternary phosphonium salts. Ordered, room-temperature ionic liquids. Chem. Mater..

[212] Ma G., Zhou Y., Su T., Wei W., Gong Y., Hu X., Hong Y., Su Y., Wang H., Li J. (2016). Phase equilibria and diffusion behavior of high pressure CO2 in tetra-n-heptyl ammonium bromide. J. Supercrit. Fluids.

[213] Domańska U., Marciniak A., Krolikowski M. (2008). Phase equilibria and modeling of ammonium ionic liquid, C2NTf2, solutions. J. Phys. Chem. B.

[214] Domanska U., Królikowski M., Ramjugernath D., Letcher T.M., Tumba K. (2010). Phase equilibria and modeling of pyridinium-based ionic liquid solutions. J. Phys. Chem. B.

[215] Vataščin E., Dohnal V. (2019). Phase equilibria of (water + [EMIM] bromide) and (water + [EMIM] tosylate). Fluid Phase Equilibria.

[216] Domańska U., Ługowska K., Pernak J. (2007). Phase equilibria of didecyldimethylammonium nitrate ionic liquid with water and organic solvents. J. Chem. Thermodyn..

[217] Arce A., Earle M.J., Katdare S.P., Rodríguez H., Seddon K.R. (2007). Phase equilibria of mixtures of mutually immiscible ionic liquids. Fluid Phase Equilibria.

[218] Wong D.S.H., Chen J.P., Chang J.M., Chou C.H. (2002). Phase equilibria of water and ionic liquids [emim][PF6] and [bmim][PF6]. Fluid Phase Equilibria.

[219] Domańska U., Królikowski M., Ślesińska K. (2009). Phase equilibria study of the binary systems (ionic liquid+thiophene): Desulphurization process. J. Chem. Thermodyn..

[220] Domanska U., Królikowski M., Pobudkowska A., Letcher T.M. (2009). Phase equilibria study of the binary systems (n-butyl-4-methylpyridinium tosylate ionic liquid+ organic solvent, or water). J. Chem. Eng. Data.

[221] Królikowski M., Królikowska M., Lipińska A. (2018). Phase equilibria study on bromide-based ionic liquids with glycols and sulfolane. Experimental data and correlation. J. Chem. Thermodyn..

[222] Crosthwaite J.M., Muldoon M.J., Dixon J.K., Anderson J.L., Brennecke J.F. (2005). Phase transition and decomposition temperatures, heat capacities and viscosities of pyridinium ionic liquids. J. Chem. Thermodyn..

[223] Ishida H., Iwachido T., Ikeda R. (1992). Phase Transitions in Dimethylammonium Tetrafluoroborate and Molecular Motions in Its Ionic Plastic Phase Studied by 1H and 19F NMR, Thermal Measurements, and X-Ray Powder Diffraction Techniques. Ber. Der Bunsenges. Für Phys. Chem..

[224] Fillion J.J., Xia H., Desilva M.A., Quiroz-Guzman M., Brennecke J.F. (2016). Phase transitions, decomposition temperatures, viscosities, and densities of phosphonium, ammonium, and imidazolium ionic liquids with aprotic heterocyclic anions. J. Chem. Eng. Data.

[225] Yeon S.-H., Kim K.-S., Choi S., Lee H., Kim H.S., Kim H. (2005). Physical and electrochemical properties of 1-(2-hydroxyethyl)-3-methyl imidazolium and N-(2-hydroxyethyl)-N-methyl morpholinium ionic liquids. Electrochim. Acta.

[226] Nishida T., Tashiro Y., Yamamoto M. (2003). Physical and electrochemical properties of 1-alkyl-3-methylimidazolium tetrafluoroborate for electrolyte. J. Fluor. Chem..

[227] Tsunashima K., Sugiya M. (2007). Physical and electrochemical properties of low-viscosity phosphonium ionic liquids as potential electrolytes. Electrochem. Commun..

[228] Matsumoto H., Kageyama H., Miyazaki Y. (2002). Physical and electrochemical properties of room temperature molten salt based on aliphatic onium cations and asymmetric amide anion. ECS Proc. Vol..

[229] Cabeza O., Vila J., Rilo E., Domínguez-Pérez M., Otero-Cernadas L., López-Lago E., Méndez-Morales T., Varela L.M. (2014). Physical properties of aqueous mixtures of the ionic 1-ethl-3-methyl imidazolium octyl sulfate: A new ionic rigid gel. J. Chem. Thermodyn..

[230] Fletcher S.I., Sillars F.B., Hudson N.E., Hall P.J. (2010). Physical properties of selected ionic liquids for use as electrolytes and other industrial applications. J. Chem. Eng. Data.

[231] González B., González E.J. (2014). Physical properties of the pure 1-methyl-1-propylpyrrolidinium bis (trifluoromethylsulfonyl) imide ionic liquid and its binary mixtures with alcohols. J. Chem. Thermodyn..

[232] Neale A.R., Schütter C., Wilde P., Goodrich P., Hardacre C., Passerini S., Balducci A., Jacquemin J. (2017). Physical–chemical characterization of binary mixtures of 1-Butyl-1-methylpyrrolidinium bis {(trifluoromethyl) sulfonyl} imide and aliphatic nitrile solvents as potential electrolytes for electrochemical energy storage applications. J. Chem. Eng. Data.

[233] Domańska U., Krolikowska M., Paduszyński K. (2011). Physico-chemical properties and phase behaviour of piperidinium-based ionic liquids. Fluid Phase Equilibria.

[234] Yamamoto T., Matsumoto K., Hagiwara R., Nohira T. (2017). Physicochemical and electrochemical properties of K [N (SO2F) 2]–[N-methyl-N-propylpyrrolidinium][N (SO2F) 2] ionic liquids for potassium-ion batteries. J. Phys. Chem. C.

[235] De La Hoz A.T., Brauer U.G., Miller K.M. (2014). Physicochemical and Thermal Properties for a Series of 1-Alkyl-4-methyl-1, 2, 4-triazolium Bis (trifluoromethylsulfonyl) imide Ionic Liquids. J. Phys. Chem. B.

[236] Zawadzki M., Królikowska M., Antonowicz J., Lipiński P., Karpińska M. (2016). Physicochemical and thermodynamic properties of the {1-alkyl-1-methylmorpholinium bromide,[C1Cn = 3, 4, 5MOR] Br, or 1-methyl-1-pentylpiperidinium bromide,[C1C5PIP] Br+ water} binary systems. J. Chem. Thermodyn..

[237] Królikowska M., Zawadzki M., Kuna T. (2019). Physicochemical and thermodynamic properties of the {1-alkyl-1-methylpiperidinium bromide [C1Cn = 2, 4PIP][Br], or 1-butylpyridinium bromide,[C4Py][Br], or tri (ethyl) butylammonium bromide [N2, 2, 2, 4][Br]+ water} binary systems. Thermochim. Acta.

[238] Ferrara C., Dall’Asta V., Berbenni V., Quartarone E., Mustarelli P. (2017). Physicochemical characterization of AlCl3–1-Ethyl-3-methylimidazolium chloride ionic liquid electrolytes for aluminum rechargeable batteries. J. Phys. Chem. C.

[239] Li H., Zhao G., Liu F., Zhang S. (2013). Physicochemical Characterization of MF m–-Based Ammonium Ionic Liquids. J. Chem. Eng. Data.

[240] González B., Gómez E., Domínguez Á., Vilas M., Tojo E. (2011). Physicochemical characterization of new sulfate ionic liquids. J. Chem. Eng. Data.

[241] Domańska U., Bogel-Łukasik R. (2005). Physicochemical properties and solubility of alkyl-(2-hydroxyethyl)-dimethylammonium bromide. J. Phys. Chem. B.

[242] Tokuda H., Hayamizu K., Ishii K., Susan M.A.B.H., Watanabe M. (2005). Physicochemical properties and structures of room temperature ionic liquids. 2. Variation of alkyl chain length in imidazolium cation. J. Phys. Chem. B.

[243] Tokuda H., Ishii K., Susan M.A.B.H., Tsuzuki S., Hayamizu K., Watanabe M. (2006). Physicochemical properties and structures of room-temperature ionic liquids. 3. Variation of cationic structures. J. Phys. Chem. B.

[244] Salminen J., Papaiconomou N., Kumar R.A., Lee J.-M., Kerr J., Newman J., Prausnitz J.M. (2007). Physicochemical properties and toxicities of hydrophobic piperidinium and pyrrolidinium ionic liquids. Fluid Phase Equilibria.

[245] Rocha M.A., van den Bruinhorst A., Schröer W., Rathke B., Kroon M.C. (2016). Physicochemical properties of fatty acid based ionic liquids. J. Chem. Thermodyn..

[246] Liu Q.-S., Yang M., Li P.-P., Sun S.-S., Welz-Biermann U., Tan Z.-C., Zhang Q.-G. (2011). Physicochemical properties of ionic liquids [C3py][NTf2] and [C6py][NTf2]. J. Chem. Eng. Data.

[247] Song Y., Liu L., Zhu X., Wang X., Jia H., Xiao X., Yu H., Yang X. (2008). Physicochemical properties of ionic liquids based on imidazolium/pyrrolidinium cations and maleate/phthalate anions. Solid State Ion..

[248] Hazrati N., Abdouss M., Miran Beigi A.A., Pasban A.A., Rezaei M. (2017). Physicochemical properties of long chain alkylated imidazolium based chloride and bis (trifluoromethanesulfonyl) imide ionic liquids. J. Chem. Eng. Data.

[249] Zhang Q., Li Z., Zhang J., Zhang S., Zhu L., Yang J., Zhang X., Deng Y. (2007). Physicochemical properties of nitrile-functionalized ionic liquids. J. Phys. Chem. B.

[250] Wei Y., Zhang Q.-G., Liu Y., Li X., Lian S., Kang Z. (2010). Physicochemical property estimation of an ionic liquid based on glutamic acid− BMIGlu. J. Chem. Eng. Data.

[251] Che Q., Sun B., He R. (2008). Preparation and characterization of new anhydrous, conducting membranes based on composites of ionic liquid trifluoroacetic propylamine and polymers of sulfonated poly (ether ether) ketone or polyvinylidenefluoride. Electrochim. Acta.

[252] Mu Z., Zhou F., Zhang S., Liang Y., Liu W. (2004). Preparation and Characterization of New Phosphonyl-Substituted Imidazolium Ionic Liquids. Helv. Chim. Acta.

[253] Branco L.C., Rosa J.N., Moura Ramos J.J., Afonso C.A. (2002). Preparation and characterization of new room temperature ionic liquids. Chem. A Eur. J..

[254] Tsukada Y., Iwamoto K., Furutani H., Matsushita Y., Abe Y., Matsumoto K., Monda K., Hayase S., Kawatsura M., Itoh T. (2006). Preparation of novel hydrophobic fluorine-substituted-alkyl sulfate ionic liquids and application as an efficient reaction medium for lipase-catalyzed reaction. Tetrahedron Lett..

[255] Matsumoto H., Sakaebe H., Tatsumi K. (2005). Preparation of room temperature ionic liquids based on aliphatic onium cations and asymmetric amide anions and their electrochemical properties as a lithium battery electrolyte. J. Power Sources.

[256] Fannin A.A., Floreani D.A., King L.A., Landers J.S., Piersma B.J., Stech D.J., Vaughn R.L., Wilkes J.S., Williams J.L. (1984). Properties of 1, 3-dialkylimidazolium chloride-aluminum chloride ionic liquids. 2. Phase transitions, densities, electrical conductivities, and viscosities. J. Phys. Chem..

[257] Fujita K., MacFarlane D.R., Forsyth M. (2005). Protein solubilising and stabilising ionic liquids. Chem. Commun..

[258] Greaves T.L., Weerawardena A., Fong C., Krodkiewska I., Drummond C.J. (2006). Protic ionic liquids: Solvents with tunable phase behavior and physicochemical properties. J. Phys. Chem. B.

[259] Canongia Lopes J.N., Esperança J.M., de Ferro A.M., Pereiro A.B., Plechkova N.V., Rebelo L.P., Seddon K.R., Vázquez-Fernández I. (2016). Protonic ammonium nitrate ionic liquids and their mixtures: Insights into their thermophysical behavior. J. Phys. Chem. B.

[260] Noda A., Hayamizu K., Watanabe M. (2001). Pulsed-gradient spin− echo 1H and 19F NMR ionic diffusion coefficient, viscosity, and ionic conductivity of non-chloroaluminate room-temperature ionic liquids. J. Phys. Chem. B.

[261] Ishikawa M., Sugimoto T., Kikuta M., Ishiko E., Kono M. (2006). Pure ionic liquid electrolytes compatible with a graphitized carbon negative electrode in rechargeable lithium-ion batteries. J. Power Sources.

[262] Macfarlane D.R., Meakin P., Sun J., Amini N., Forsyth M. (1999). Pyrrolidinium imides: A new family of molten salts and conductive plastic crystal phases. J. Phys. Chem. B.

[263] Singh R.P., Winter R.W., Gard G.L., Gao Y., Shreeve J.M. (2003). Quaternary salts containing the pentafluorosulfanyl (SF5) group. Inorg. Chem..

[264] Earle M.J., McCormac P.B., Seddon K.R. (1998). Regioselective alkylation in ionic liquids. Chem. Commun..

[265] Gu Y.-L., Shi F., Deng Y.-Q. (2004). Room temperature ionic liquid as leaching reagent for separation of the solid mixture of taurine and sodium sulfate. Acta Chim. Sin. Chin. Ed..

[266] Matsumoto K., Hagiwara R., Ito Y. (2002). Room temperature molten fluorometallates: 1-ethyl-3-methylimidazolium hexafluoroniobate(V) and hexafluorotantalate(V). J. Fluor. Chem..

[267] Matsumoto H., Kageyama H., Miyazaki Y. (2001). Room temperature molten salts based on tetraalkylammonium cations and bis (trifluoromethylsulfonyl) imide. Chem. Lett..

[268] Matsumoto H., Matsuda T., Miyazaki Y. (2000). Room temperature molten salts based on trialkylsulfonium cations and bis (trifluoromethylsulfonyl) imide. Chem. Lett..

[269] Liu Q., Janssen M.H., van Rantwijk F., Sheldon R.A. (2005). Room-temperature ionic liquids that dissolve carbohydrates in high concentrations. Green Chem..

[270] Sun J., Forsyth M., Macfarlane D.R. (1998). Room-temperature molten salts based on the quaternary ammonium ion. J. Phys. Chem. B.

[271] McMichael K., Clement R. (1961). Salt effects in the solvolysis of benzhydryl chloride. J. Org. Chem..

[272] Wlazło M., Zawadzki M., Domańska U. (2018). Separation of water/butan-1-ol based on activity coefficients at infinite dilution in 1, 3-didecyl-2-methylimidazolium dicyanamide ionic liquid. J. Chem. Thermodyn..

[273] Bradley A.E., Hardacre C., Holbrey J.D., Johnston S., McMath S.E.J., Nieuwenhuyzen M. (2002). Small-angle X-ray scattering studies of liquid crystalline 1-alkyl-3-methylimidazolium salts. Chem. Mater..

[274] Sifaoui H., Ait-Kaci A., Modarressi A., Rogalski M. (2007). Solid–liquid equilibria of three binary systems:{1-Ethyl-3-methylimidazolium hexafluorophosphate+ 2-phenylimidazole, or 4,5-diphenylimidazole or 2,4,5-triphenylimidazole}. Thermochim. Acta.

[275] Kick M., Keil P., König A. (2013). Solid–liquid phase diagram of the two Ionic Liquids EMIMCl and BMIMCl. Fluid Phase Equilibria.

[276] Shevelyova M.P., Zaitsau D.H., Paulechka Y.U., Blokhin A.V., Kabo G.J., Verevkin S.P., Heintz A. (2007). Solid− Liquid Equilibrium and Activity Coefficients for Caprolactam+ 1-Hexyl-3-methylimidazolium Bis (trifluoromethylsulfonyl) imide and Cyclohexanone Oxime+ 1-Hexyl-3-methylimidazolium Bis (trifluoromethylsulfonyl) imide. J. Chem. Eng. Data.

[277] Yang X.-Z., Wang J., Li G.-S., Zhang Z.-Z. (2009). Solubilities of 1-ethylpyridinium hexafluorophosphate in ethanol+ water from (278.15 to 345.15) K. J. Chem. Eng. Data.

[278] Lourenço C., Melo C.I., Bogel-Łukasik R., Bogel-Łukasik E. (2012). Solubility advantage of pyrazine-2-carboxamide: Application of alternative solvents on the way to the future pharmaceutical development. J. Chem. Eng. Data.

[279] Domanska U., Rekawek A., Marciniak A. (2008). Solubility of 1-alkyl-3-ethylimidazolium-based ionic liquids in water and 1-octanol. J. Chem. Eng. Data.

[280] Domańska U., Marciniak A. (2003). Solubility of 1-alkyl-3-methylimidazolium hexafluorophosphate in hydrocarbons. J. Chem. Eng. Data.

[281] Domańska U., Bogel-Łukasik E., Bogel-Łukasik R. (2003). Solubility of 1-Dodecyl-3-methylimidazolium Chloride in Alcohols (C2− C12). J. Phys. Chem. B.

[282] Domańska U., Mazurowska L. (2004). Solubility of 1, 3-dialkylimidazolium chloride or hexafluorophosphate or methylsulfonate in organic solvents: Effect of the anions on solubility. Fluid Phase Equilibria.

[283] Langham J.V., O’Brien R.A., Davis J.H., West K.N. (2016). Solubility of CO2 and N2O in an imidazolium-based lipidic ionic liquid. J. Phys. Chem. B.

[284] Ramdin M., Vlugt T.J., de Loos T.W. (2012). Solubility of CO2 in the ionic liquids [TBMN][MeSO4] and [TBMP][MeSO4]. J. Chem. Eng. Data.

[285] Okuniewska P., Ramjugernath D., Naidoo P., Domańska U. (2014). Solubility of ionic liquids in 2-phenylethanol (PEA) and water. Fluid Phase Equilibria.

[286] Neves C.M., Rodrigues A.R., Kurnia K.A., Esperança J.M., Freire M.G., Coutinho J.A. (2013). Solubility of non-aromatic hexafluorophosphate-based salts and ionic liquids in water determined by electrical conductivity. Fluid Phase Equilibria.

[287] Domańska U., Casás L.M. (2007). Solubility of Phosphonium Ionic Liquid in Alcohols, Benzene, and Alkylbenzenes. J. Phys. Chem. B.

[288] Sarkar A., Sinha B. (2013). Solution thermodynamics of aqueous nicotinic acid solutions in presence of tetrabutylammonium hydrogen sulphate. J. Serb. Chem. Soc..

[289] Sarkar A., Mishra D.K., Sinha B. (2016). Solution thermophysics of l-ascorbic acid in aqueous tetrabutylammonium hydrogen sulfate. J. Solut. Chem..

[290] Katsuta S., Shiozawa Y., Imai K., Kudo Y., Takeda Y. (2010). Stability of ion pairs of bis (trifluoromethanesulfonyl) amide-based ionic liquids in dichloromethane. J. Chem. Eng. Data.

[291] MacFarlane D.R., Meakin P., Amini N., Forsyth M. (2001). Structural studies of ambient temperature plastic crystal ion conductors. J. Phys. Condens. Matter.

[292] Zhang Q.-G., Xue F., Tong J., Guan W., Wang B. (2006). Studies on volumetric properties of concentrated aqueous solutions of the ionic liquid BMIBF 4. J. Solut. Chem..

[293] Studzińska S., Sprynskyy M., Buszewski B. (2008). Study of sorption kinetics of some ionic liquids on different soil types. Chemosphere.

[294] Yang J.-Z., Tong J., Li J.-B. (2007). Study of the volumetric properties of the aqueous ionic liquid 1-methyl-3-pentylimidazolium tetrafluoroborate. J. Solut. Chem..

[295] Law G., Watson P.R. (2001). Surface tension measurements of N-alkylimidazolium ionic liquids. Langmuir.

[296] Pernak J., Wasiński K., Praczyk T., Nawrot J., Cieniecka-Rosłonkiewicz A., Walkiewicz F., Materna K. (2012). Sweet ionic liquids-cyclamates: Synthesis, properties, and application as feeding deterrents. Sci. China Chem..

[297] Carter E.B., Culver S.L., Fox P.A., Goode R.D., Ntai I., Tickell M.D., Traylor R.K., Hoffman N.W., Davis J.H. (2004). Sweet success: Ionic liquids derived from non-nutritive sweeteners. Chem. Commun..

[298] Mirzaei Y.R., Twamley B., Shreeve J.M. (2002). Syntheses of 1-alkyl-1,2,4-triazoles and the formation of quaternary 1-alkyl-4-polyfluoroalkyl-1, 2, 4-triazolium salts leading to ionic liquids. J. Org. Chem..

[299] Ye C., Shreeve J.M. (2004). Syntheses of very dense halogenated liquids. J. Org. Chem..

[300] Matsumoto K., Hagiwara R., Yoshida R., Ito Y., Mazej Z., Benkič P., Žemva B., Tamada O., Yoshino H., Matsubara S. (2004). Syntheses, structures and properties of 1-ethyl-3-methylimidazolium salts of fluorocomplex anions. Dalton Trans..

[301] Pereira M.M., Pedro S.N., Gomes J., Sintra T.E., Ventura S.P., Coutinho J.A., Freire M.G., Mohamadou A. (2019). Synthesis and characterization of analogues of glycine-betaine ionic liquids and their use in the formation of aqueous biphasic systems. Fluid Phase Equilibria.

[302] Zhao D., Fei Z., Scopelliti R., Dyson P.J. (2004). Synthesis and characterization of ionic liquids incorporating the nitrile functionality. Inorg. Chem..

[303] Suarez P.A.Z., Einloft S., Dullius J.E.L., de Souza R.F., Dupont J. (1998). Synthesis and physical-chemical properties of ionic liquids based on 1-n-butyl-3-methylimidazolium cation. J. Chim. Phys..

[304] Engin Özdil S., Akbaş H., Boz M. (2016). Synthesis and physicochemical properties of double-chain cationic surfactants. J. Chem. Eng. Data.

[305] Quek S.K., Lyapkalo I.M., Huynh H.V. (2006). Synthesis and properties of N, N′-dialkylimidazolium bis (nonafluorobutane-1-sulfonyl) imides: A new subfamily of ionic liquids. Tetrahedron.

[306] Papaiconomou N., Yakelis N., Salminen J., Bergman R., Prausnitz J.M. (2006). Synthesis and properties of seven ionic liquids containing 1-methyl-3-octylimidazolium or 1-butyl-4-methylpyridinium cations. J. Chem. Eng. Data.

[307] Dong L., Zheng D.X., Wei Z., Wu X.H. (2009). Synthesis of 1, 3-dimethylimidazolium chloride and volumetric property investigations of its aqueous solution. Int. J. Thermophys..

[308] Xiao J.-C., Shreeve J.M. (2005). Synthesis of 2, 2′-biimidazolium-based ionic liquids: Use as a new reaction medium and ligand for palladium-catalyzed suzuki cross-coupling reactions. J. Org. Chem..

[309] Bao W., Wang Z., Li Y. (2003). Synthesis of chiral ionic liquids from natural amino acids. J. Org. Chem..

[310] Tseng M.-C., Liang Y.-M., Chu Y.-H. (2005). Synthesis of fused tetrahydro-β-carbolinequinoxalinones in 1-n-butyl-2, 3-dimethylimidazolium bis (trifluoromethylsulfonyl) imide ([bdmim][Tf2N]) and 1-n-butyl-2, 3-dimethylimidazolium perfluorobutylsulfonate ([bdmim][PFBuSO3]) ionic liquids. Tetrahedron Lett..

[311] Wang Z., Wang Q., Zhang Y., Bao W. (2005). Synthesis of new chiral ionic liquids from natural acids and their applications in enantioselective Michael addition. Tetrahedron Lett..

[312] Dorn S., Pfeifer A., Schlufter K., Heinze T. (2010). Synthesis of water-soluble cellulose esters applying carboxylic acid imidazolides. Polym. Bull..

[313] Cieniecka-Rosłonkiewicz A., Pernak J., Kubis-Feder J., Ramani A., Robertson A.J., Seddon K.R. (2005). Synthesis, anti-microbial activities and anti-electrostatic properties of phosphonium-based ionic liquids. Green Chem..

[314] Ding Y.-S., Zha M., Zhang J., Wang S.-S. (2007). Synthesis, characterization and properties of geminal imidazolium ionic liquids. Colloids Surf. A Physicochem. Eng. Asp..

[315] Busi S., Lahtinen M., Valkonen J., Rissanen K. (2008). Synthesis, characterization and thermal behavior of nine new R2R2′ N^+^A^−^-type quaternary ammonium tetrafluoroborate or hexafluorophosphate salts prepared by metathesis from analogous halide salts. J. Mol. Struct..

[316] Busi S., Lahtinen M., Mansikkamäki H., Valkonen J., Rissanen K. (2005). Synthesis, characterization and thermal properties of small R2R′ 2N+ X−-type quaternary ammonium halides. J. Solid State Chem..

[317] Kölle P., Dronskowski R. (2004). Synthesis, crystal structures and electrical conductivities of the ionic liquid compounds butyldimethylimidazolium tetrafluoroborate, hexafluorophosphate and hexafluoroantimonate. Eur. J. Inorg. Chem..

[318] Marciniak A., Królikowski M. (2012). Ternary (liquid+ liquid) equilibria of {trifluorotris (perfluoroethyl) phosphate based ionic liquids+ thiophene+ heptane}. J. Chem. Thermodyn..

[319] Pringle J.M., Golding J., Baranyai K., Forsyth C.M., Deacon G.B., Scott J.L., MacFarlane D.R. (2003). The effect of anion fluorination in ionic liquids—physical properties of a range of bis (methanesulfonyl) amide salts. New J. Chem..

[320] Brauer U.G., Andreah T., Miller K.M. (2015). The effect of counteranion on the physicochemical and thermal properties of 4-methyl-1-propyl-1, 2, 4-triazolium ionic liquids. J. Mol. Liq..

[321] Rodrigues A.S., Almeida H.F., Freire M.G., Lopes-da-Silva J.A., Coutinho J.A., Santos L.M. (2016). The effect of n vs. iso isomerization on the thermophysical properties of aromatic and non-aromatic ionic liquids. Fluid Phase Equilibria.

[322] Bagno A., Butts C., Chiappe C., D’Amico F., Lord J.C., Pieraccini D., Rastrelli F. (2005). The effect of the anion on the physical properties of trihalide-based N, N-dialkylimidazolium ionic liquids. Org. Biomol. Chem..

[323] Krolikowska M., Zawadzki M., Skonieczny M. (2018). The influence of bromide-based ionic liquids on solubility of {LiBr (1)+ water (2)} system. Experimental (solid+ liquid) phase equilibrium data. Part 2. J. Mol. Liq..

[324] Holbrey J.D., Seddon K.R. (1999). The phase behaviour of 1-alkyl-3-methylimidazolium tetrafluoroborates; ionic liquids and ionic liquid crystals. J. Chem. Soc. Dalton Trans..

[325] Bogel-Łukasik R., Matkowska D., Zakrzewska M.E., Bogel-Łukasik E., Hofman T. (2010). The phase envelopes of alternative solvents (ionic liquid, CO2) and building blocks of biomass origin (lactic acid, propionic acid). Fluid Phase Equilibria.

[326] Fuller J., Carlin R.T., Osteryoung R.A. (1997). The room temperature ionic liquid 1-ethyl-3-methylimidazolium tetrafluoroborate: Electrochemical couples and physical properties. J. Electrochem. Soc..

[327] Alves M.B., Umpierre A.P., Santos V.O., Soares V.C.D., Dupont J., Rubim J.C., Suarez P.A.Z. (2010). The use of Differential Scanning Calorimetry (DSC) to characterize phase diagrams of ionic mixtures of 1-n-butyl-3-methylimidazolium chloride and niobium chloride or zinc chloride. Thermochim. Acta.

[328] Gómez E., Calvar N., Domínguez Á., Macedo E.A. (2013). Thermal analysis and heat capacities of 1-Alkyl-3-methylimidazolium ionic liquids with NTf_2_^−^, TFO^−^, and DCA^−^ anions. Ind. Eng. Chem. Res..

[329] Calvar N., Gómez E., Macedo E.A., Domínguez Á. (2013). Thermal analysis and heat capacities of pyridinium and imidazolium ionic liquids. Thermochim. Acta.

[330] Mutch M.L., Wilkes J.S. (1998). Thermal analysis of 1-ethyl-3-methylimidazolium tetrafluoroborate molten salt. ECS Proc. Vol..

[331] Nemoto F., Kofu M., Yamamuro O. (2015). Thermal and structural studies of imidazolium-based ionic liquids with and without liquid-crystalline phases: The origin of nanostructure. J. Phys. Chem. B.

[332] Gómez E., Calvar N., Domínguez Á., Macedo E.A. (2018). Thermal behavior and heat capacities of pyrrolidinium-based ionic liquids by DSC. Fluid Phase Equilibria.

[333] Stolarska O., Soto A., Rodríguez H., Smiglak M. (2018). Thermal behaviour of mixtures of 1-alkylpyridinium halides with and without a common ion. J. Mol. Liq..

[334] Ngo H.L., LeCompte K., Hargens L., McEwen A.B. (2000). Thermal properties of imidazolium ionic liquids. Thermochim. Acta.

[335] Efimova A., Hubrig G., Schmidt P. (2013). Thermal stability and crystallization behavior of imidazolium halide ionic liquids. Thermochim. Acta.

[336] Monteiro B., Maria L., Cruz A., Carretas J.M., Marçalo J., Leal J.P. (2020). Thermal stability and specific heats of coordinating ionic liquids. Thermochim. Acta.

[337] Guan W., Yang J.-Z., Li L., Wang H., Zhang Q.-G. (2006). Thermo-chemical properties of aqueous solution containing ionic liquids: 1. The heat of reaction mixed 1-methyl-3-butylimidazolium chloride with InCl3. Fluid Phase Equilibria.

[338] Jagadeeswara Rao C., Venkata Krishnan R., Venkatesan K., Nagarajan K., Srinivasan T. (2009). Thermochemical properties of some bis (trifluoromethyl-sulfonyl) imide based room temperature ionic liquids. J. Therm. Anal. Calorim..

[339] Tong B., Liu Q.-S., Tan Z.-C., Welz-Biermann U. (2010). Thermochemistry of alkyl pyridinium bromide ionic liquids: Calorimetric measurements and calculations. J. Phys. Chem. A.

[340] Van Valkenburg M.E., Vaughn R.L., Williams M., Wilkes J.S. (2005). Thermochemistry of ionic liquid heat-transfer fluids. Thermochim. Acta.

[341] Zhang Z., Sun L., Tan Z., Xu F., Lv X., Zeng J., Sawada Y. (2007). Thermodynamic investigation of room temperature ionic liquid: Heat capacity and thermodynamic functions of BPBF4. J. Therm. Anal. Calorim..

[342] Domańska U., Żołek-Tryznowska Z., Królikowski M. (2007). Thermodynamic Phase Behavior of Ionic Liquids. J. Chem. Eng. Data.

[343] Archer D.G. (2006). Thermodynamic Properties of 1-hexyl-3-methylimidazolium Bis (trifluoromethylsulfony) Imide.

[344] Allen M., Evans D.F., Lumry R. (1985). Thermodynamic properties of the ethylammonium nitrate+ water system: Partial molar volumes, heat capacities, and expansivities. J. Solut. Chem..

[345] Domańska U., Królikowski M., Acree W.E. (2011). Thermodynamics and activity coefficients at infinite dilution measurements for organic solutes and water in the ionic liquid 1-butyl-1-methylpyrrolidinium tetracyanoborate. J. Chem. Thermodyn..

[346] Domańska U., Wlazło M. (2016). Thermodynamics and limiting activity coefficients measurements for organic solutes and water in the ionic liquid 1-dodecyl-3-methylimidzolium bis (trifluoromethylsulfonyl) imide. J. Chem. Thermodyn..

[347] Sakizadeh K., Olson L.P., Cowdery-Corvan P.J., Ishida T., Whitcomb D.R. (2007). Thermographic Materials Containing Ionic Liquids. U.S. Patent.

[348] García-Andreu M., Castro M., Gascón I., Lafuente C. (2016). Thermophysical characterization of 1-ethylpyridinium triflate and comparison with similar ionic liquids. J. Chem. Thermodyn..

[349] Neves C.M., Batista M.L., Cláudio A.F.M., Santos L.M., Marrucho I.M., Freire M.G., Coutinho J.A. (2010). Thermophysical properties and water saturation of [PF6]-based ionic liquids. J. Chem. Eng. Data.

[350] Chhotaray P.K., Gardas R.L. (2014). Thermophysical properties of ammonium and hydroxylammonium protic ionic liquids. J. Chem. Thermodyn..

[351] Parajo J.J., Villanueva M., Troncoso J., Salgado J. (2020). Thermophysical properties of choline and pyridinium based ionic liquids as advanced materials for energy applications. J. Chem. Thermodyn..

[352] Zhang H., Xu W., Liu J., Li M., Yang B. (2019). Thermophysical properties of dicationic imidazolium-based ionic compounds for thermal storage. J. Mol. Liq..

[353] Govinda V., Attri P., Venkatesu P., Venkateswarlu P. (2011). Thermophysical properties of dimethylsulfoxide with ionic liquids at various temperatures. Fluid Phase Equilibria.

[354] Fredlake C.P., Crosthwaite J.M., Hert D.G., Aki S.N., Brennecke J.F. (2004). Thermophysical properties of imidazolium-based ionic liquids. J. Chem. Eng. Data.

[355] Tao R., Tamas G., Xue L., Simon S.L., Quitevis E.L. (2014). Thermophysical properties of imidazolium-based ionic liquids: The effect of aliphatic versus aromatic functionality. J. Chem. Eng. Data.

[356] Murray S.M., Zimlich T.K., Mirjafari A., O’Brien R.A., Davis J.H., West K.N. (2013). Thermophysical properties of imidazolium-based lipidic ionic liquids. J. Chem. Eng. Data.

[357] Sudholter E.J.R., Engberts J.B.F.N., de Jeu W.H. (1981). Thermotropic Liquid-Crystallhe Behavior of Some Single- and Double-Chalned PyrMinium Amphlphlles. J. Phys. Chem..

[358] Zarrougui R., Dhahbi M., Lemordant D. (2015). Transport and thermodynamic properties of ethylammonium nitrate–water binary mixtures: Effect of temperature and composition. J. Solut. Chem..

[359] Gerhard D., Alpaslan S.C., Gores H.J., Uerdingen M., Wasserscheid P. (2005). Trialkylsulfonium dicyanamides-a new family of ionic liquids with very low viscosities. Chem. Commun..

[360] Shmukler L.E., Gruzdev M.S., Kudryakova N.O., Fadeeva Y.A., Kolker A.M., Safonova L.P. (2018). Triethylammonium-based protic ionic liquids with sulfonic acids: Phase behavior and electrochemistry. J. Mol. Liq..

[361] Grishina E.P., Ramenskaya L.M., Gruzdev M.S., Kraeva O.V. (2013). Water effect on physicochemical properties of 1-butyl-3-methylimidazolium based ionic liquids with inorganic anions. J. Mol. Liq..

[362] McKinney W., van der Walt S., Millman J. (2010). Data Structures for Statistical Computing in Python. Proceedings of the 9th Python in Science Conference.

[363] Waskom M. (2021). Seaborn: Statistical Data Visualization. https://github.com/seaborn.

[364] Hunter J.D. (2007). Matplotlib: A 2D Graphics Environment. Comput. Sci. Eng..

[365] Chollet F. (2015). Keras; GitHub Repository. https://github.com/keras-team/keras.

